# Predicting wildlife reservoirs and global vulnerability to zoonotic *Flaviviruses*

**DOI:** 10.1038/s41467-018-07896-2

**Published:** 2018-12-21

**Authors:** Pranav S. Pandit, Megan M. Doyle, Katrina M. Smart, Cristin C. W. Young, Gaylen W. Drape, Christine K. Johnson

**Affiliations:** 1 EpiCenter for Disease Dynamics, One Health Institute, School of Veterinary Medicine, University of California Davis, One Shields Ave, CA 95616 USA; 2 ENSCO Inc., 4849 North Wickham Road, Melbourne, FL 32940 USA

## Abstract

Flaviviruses continue to cause globally relevant epidemics and have emerged or re-emerged in regions that were previously unaffected. Factors determining emergence of flaviviruses and continuing circulation in sylvatic cycles are incompletely understood. Here we identify potential sylvatic reservoirs of flaviviruses and characterize the macro-ecological traits common to known wildlife hosts to predict the risk of sylvatic flavivirus transmission among wildlife and identify regions that could be vulnerable to outbreaks. We evaluate variability in wildlife hosts for zoonotic flaviviruses and find that flaviviruses group together in distinct clusters with similar hosts. Models incorporating ecological and climatic variables as well as life history traits shared by flaviviruses predict new host species with similar host characteristics. The combination of vector distribution data with models for flavivirus hosts allows for prediction of  global vulnerability to flaviviruses and provides potential targets for disease surveillance in animals and humans.

## Abstract (web summary)

Flaviviruses have emerged or re-emerged in several regions, but factors underlying emergence are incompletely understood. Here, Pandit et al. identify potential sylvatic reservoirs of flaviviruses and, in combination with vector distribution data, predict regions of global vulnerability.

## Introduction

The recent emergence of Zika virus (ZIKV) across South America and the spread of West Nile virus (WNV) in North America in the early 2000s reflect the increasing public health risk and pandemic potential posed by flaviviruses globally^1^. Flaviviruses are transmitted among a wide variety of mammalian and avian hosts, with sylvatic cycles that perpetuate new outbreaks through spillover into humans and hinder disease prevention and control efforts. Migratory birds are hypothesized to have spread WNV in temperate areas of the Old and New World, and human WNV outbreaks in North America have been associated with corresponding WNV outbreaks in wild birds^2,3^. Flaviviruses such as yellow fever virus (YFV) and Japanese encephalitis virus (JEV) have well-recognized sylvatic cycles that maintain viral circulation in wild reservoirs between outbreaks in human populations^4^. Therefore, improved understanding of the potential wildlife host involvement in the transmission of emerging flaviviruses is essential. For instance, data about the transmission of ZIKV in South American wildlife are critical to inform on maintenance of the virus in the region, as sylvatic hosts can serve as a source of recurring epidemics and impede efforts for long-term disease prevention^5^.

Surveillance programs which aim to identify sylvatic hosts of viral diseases face many logistical hurdles, including accessing wild species that often reside in remote habitats and procuring large numbers of high-quality samples for virus detection. However, recognition of wildlife hosts for zoonotic pathogens is important for evaluating pathogen evolution within wildlife reservoir populations, advancing early detection of outbreaks through identification of sentinel species, mitigating risk at animal–human interfaces, and monitoring threats that zoonotic pathogens might pose to wildlife^6^. When compared to viruses with a limited host range, zoonotic viruses with high host plasticity are more likely to spread after spillover by secondary human-to-human transmission and are known to have greater geographic spread^7^. While vector-borne viruses have greater host plasticity in general^7^, members of the flavivirus genus demonstrate a varying propensity towards host range; some infect species within a narrow range of taxonomic relatedness while others infect a wide range of vertebrate taxa^4^.

In addition to viral traits^8^, host and environmental factors such as the ecological niche of reservoir hosts, host interactions with potential vectors, and seasonal and climatic conditions play a significant role in sustaining viral persistence and evolution in sylvatic host populations^9^. Identifying macro-ecological patterns that facilitate viral persistence is crucial for understanding global variation in prevalence and propensity of flavivirus for epidemiologically important sylvatic cycles. Here, we collect data from published reports of non-human vertebrate hosts for thirty-five zoonotic flaviviruses (66% of the species within the genus *Flavivirus*^10^) known to cause human infection and associated with at least one non-human vertebrate host. We characterize the environmental and life history traits of known flavivirus hosts and identify new hosts whose trait profiles indicate a high probability for harboring flaviviruses. Our results identify regions with a high number of potential flavivirus hosts and vulnerability to sylvatic transmission.

## Results

### Mammalian and avian hosts of *Flaviviruses*

**Fig. 1:**
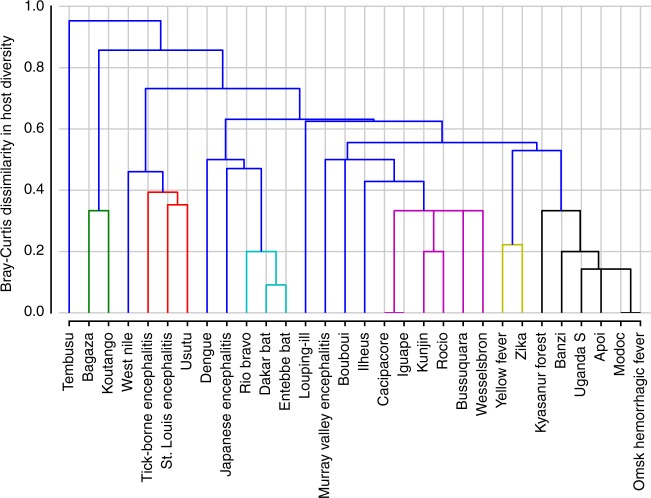
Co-occurrence of avian and mammalian hosts among Flaviviruses. Cluster analysis of zoonotic flaviviruses according to known host taxonomic orders and their Bray-Curtis dissimilarity index. Green, red, cyan, purple, yellow, and black lines show clusters of viruses with similar host taxonomic orders (Bray-Curtis dissimilarity index < 0.4)

Among thirty-five zoonotic flaviviruses, we identified thirty flaviviruses with non-human vertebrate hosts reported with natural flavivirus infection confirmed by either virus isolation, polymerase chain reaction (PCR), or plaque reduction neutralization test (PRNT). We detected 140 mammals (out of 5,536 mammal species^11^) and 277 avian species (out of 10,424 avian species^12^) as recognized hosts for flaviviruses. Flaviviruses were found to have an average of 18.9 hosts with a median of 4 hosts. Taken together, flaviviruses had a wide range of propensity for host plasticity based on diversity of species discovered to date, ranging from four flaviviruses (Bouboui virus, Cacipacore virus, Iguape virus, and Koutango virus) with only one host species, to WNV with a maximum of 194 host species recognized to date (Table 1, Supplementary Data file 1). Cluster analyses of zoonotic flaviviruses revealed multiple prominent clusters of viruses which share hosts from similar taxonomical orders. Group 1 viruses (ZIKV, YFV) were predominantly found in primate hosts. Group 2 viruses, which included WNV, St. Louis encephalitis virus (SLEV), Usutu virus (USUV), and tick-borne encephalitis virus (TBEV), were found in a variety of birds and mammalian orders. Lastly, Group 3 viruses included Rio Bravo virus (RBV), Dakar bat virus (DBV), and Entebbe bat virus (ENTV) that were exclusive to bats. Japanese encephalitis virus (JEV) and dengue virus (DENV) did not cluster with any other flaviviruses (Fig. 1).

**Table 1:** Number of known flavivirus host by virus,  classified by Order and Family showing a range of host plasticity in the flavivirus genus

Virus	Order	Family	Species
Apoi virus	1	2	4
Bagaza virus	1	1	2
Banzi virus	1	2	2
Bouboui virus	1	1	1
Bussuquara virus	3	3	3
Cacipacore virus	1	1	1
Dakar bat virus	1	3	5
Dengue virus	5	11	27
Entebbe bat virus	1	4	6
Iguape virus	1	1	1
Ilheus virus	3	4	5
Japanese encephalitis virus	7	13	25
Koutango virus	1	1	1
Kunjin virus	3	3	3
Kyasanur forest virus	3	3	6
Louping-ill virus	7	9	13
Modoc virus	1	2	3
Murray valley encephalitis virus	2	2	3
Omsk hemorrhagic fever virus	1	1	3
Rio Bravo virus	1	3	9
Rocio virus	2	2	2
St. Louis encephalitis virus	19	39	72
Tembusu virus	1	1	2
Tick-borne encephalitis virus	14	33	83
Uganda S virus	2	3	4
Usutu virus	17	31	65
Wesselsbron virus	3	3	3
West Nile virus	29	72	194
Yellow fever virus	2	6	11
Zika virus	1	4	7

### Macroecological traits of flaviviral hosts

We constructed a generic model using the generalized boosted regression tree method for all flaviviruses and developed three stratified models based on virus cluster Groups 1, 2, and 3 and independent models for TBEV, JEV, and DENV to predict if a species is a flaviviruses host (or host of one of the viruses from the groups). Although TBEV shared hosts similar to WNV, SLEV, and USUV, it was modeled separately as it is transmitted by ticks while the other viruses are transmitted by mosquitoes. The optimal number of recursive trees where the holdout deviance was minimized in our models varied from 1,250 to 11,200 trees. Details of the number of trees, cross-validation AUC, and holdout AUC are shown in Supplementary Table 1.

**Fig. 2:**
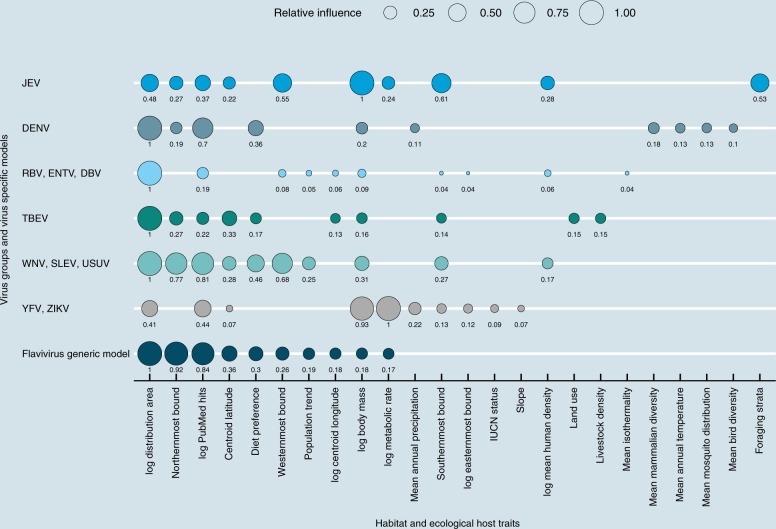
Macro-ecological traits of flavivirus hosts. Plot describing the ten most important biological and ecological host traits and their relative influence for all models predicting flavivirus hosts. YFV, yellow fever virus; ZIKV, Zika virus; WNV, West Nile virus; SLEV, St. Louis encephalitis virus; USUV, Usutu virus; TBEV, Tick-borne encephalitis virus; RBV, Rio Bravo virus; ENTV, Entebbe bat virus; DBV, Dakar bat virus; DENV, dengue virus; JEV, Japanese encephalitis virus

The generic model for all flaviviruses revealed the relative importance of 29 host traits related to host ecology, conservation status, physiology, geographical distribution, vector distribution, climatic variables, anthropogenic variables, and bird and mammalian diversity within the host range that influenced the probability of mammalian and avian species hosting flaviviruses, with a cross-validation AUC of 0.906 (Supplementary Table 1). The variable describing the northernmost latitude of host species distribution (northernmost bound), showed the highest relative influence for this generic model followed by the geographical range of the species (log distribution area km^2^). The number of PubMed hits for species was used to control for sampling effort and reporting bias, was found to be the third most important variable. When the generic model was run both including and excluding number of PubMed hits, nine out of the top ten variables were retained as significant, indicating that although some species are well-studied, sampling effort did not substantially bias the overall results and interpretation for important macro-ecological traits of flavivirus hosts. With the exclusion of number of PubMed hits, westernmost bound of species distribution, metabolic rate, and body mass gained relative variable importance and rank, while the variables related to species population trend and latitude of species distribution centroid went down in the ranking. Southernmost bound was the new variable introduced into the top ten variables, which was otherwise ranked 12^th^ for the generic model (Supplementary Figure 1, Supplementary Figure 2 and Supplementary Data file 2). The trait profile of flavivirus-positive hosts indicated that hosts tend to have a wider geographic range, reside in more isothermal regions (between latitudes of 10°N–40°N) compared to non-host species, and have range distributions centered in subtropical regions, (Fig. 2, Supplementary Figure 1, Supplementary Figure 3). In addition to northernmost latitude bound, southernmost and westernmost longitude bounds of host distribution were also found to be influential in multiple models. Host body mass (g) and metabolic rate for flavivirus host species showed a bimodal pattern, and species with scavenging habits showed a greater likelihood of hosting flaviviruses. The family *Atelidae* (New World monkeys, including howler, spider, woolly, and woolly spider monkeys) showed the highest relative influence for hosting flaviviruses compared to other families with respect to its influence on overall model predictions (Supplementary Data file 2). Additionally, an increase in the relative probability of a group of mosquitos and ticks within the host distribution range (12th and 14th influential variables respectively), was associated with higher probability of mammalian and bird species hosting flaviviruses (Supplementary Data file 2, Supplementary Figure 4). Results of the generic model were heavily influenced by a large number of avian hosts reported for WNV, SLEV, and USUV. Overall, the geographical range of the species, host body mass (g), and the number of PubMed hits for the hosts were among the top ten most influential variables in all independent and virus group models (Fig. 2).

Model for YFV and ZIKV (Group 1 model) revealed the influence of all 29 host traits had on hosting Group 1 viruses and did not detect any taxonomical clustering within Primates. Hosts for YFV and ZIKV from orders Primates and Rodentia were heavier in mass, had higher metabolic rates, and had a wider distribution area with higher annual precipitation within their geographical distribution than non-host species (Supplementary Figure 5). Critically endangered Primate species were more likely to be hosts for Group 1 viruses than species with other IUCN Red list classification status (Supplementary Figure 5).

The predictive model for Group 2 viruses (WNV, SLEV, USUV) primarily found in avian hosts showed that species positive for these viruses had similar geographic distribution, with five out of the top ten variables related to geographic distribution (Fig. 2, Supplementary Figure 6). Host species had larger distribution areas generally centered around the Nearctic region compared to non-host species. Species were clustered in the avian families *Accipitridae*, *Mimidae*, and *Columbidae* which had the highest relative influence compared to all other families (Supplementary Data file 2).

Within Chiroptera, species positive for bat-specific flaviviruses RBV, ENTV, and DBV (Group 3) were found to be heavier in weight and residing in regions with higher human density (Fig. 2, Supplementary Figure 7). Model results for TBEV showed that body mass and diet preference influenced the likelihood of being a host for TBEV. Likelihood of being a host for TBEV was also influenced by anthropogenic factors, such land use type and density of livestock within the host distribution (Fig. 2, Supplementary Figure 8). Body mass of species showed a bimodal pattern for its partial dependence on the likelihood of being DENV hosts. DENV hosts were found to be living in areas with higher mammalian and avian diversity and with a high probability of mosquito presence (Supplementary Figure 9). JEV hosts were found in regions with higher human population density and in species that had a higher metabolic rate than non-host species (Supplementary Figure 10).

### Identification and distribution of novel flaviviral hosts

**Fig. 3:**
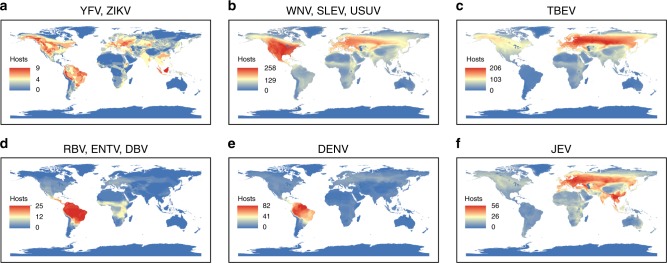
Geographical distribution of predicted flaviviral host richness. Overlapping geographical ranges of model predicted sylvatic hosts in the 95th percentile of probability for stratified models. **a** Yellow fever virus (YFV) and Zika virus (ZIKV). **b** West Nile virus (WNV), St. Louis encephalitis virus (SLEV) and Usutu virus (USUV). **c** Tick-borne encephalitis virus (TBEV). **d** Rio Bravo virus (RBV), Entebbe bat virus (ENTV) and Dakar bat virus (DBV). **e** Dengue virus (DENV). **f** Japanese encephalitis virus (JEV). Maps were generated using species distribution data from IUCN^12^, and BirdLife International and NatureServe^60^

**Fig. 4:**
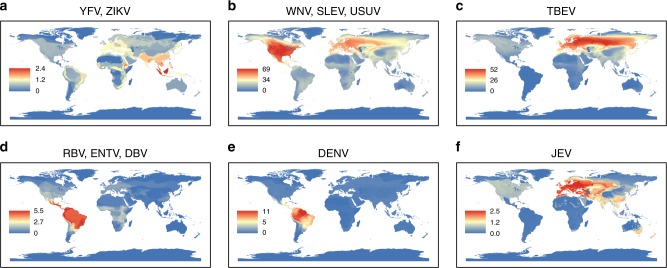
Geographical distribution of predicted flaviviral host richness adjusted by the predicted probability. Overlapping geographical ranges accounting for associated probabilities for model predicted hosts in the 95th percentile of probability for each stratified model. **a** Yellow fever virus (YFV) and Zika virus (ZIKV). **b** West Nile virus (WNV), St. Louis encephalitis virus (SLEV) and Usutu virus (USUV). **c** Tick-borne encephalitis virus (TBEV). **d** Rio Bravo virus (RBV), Entebbe bat virus (ENTV) and Dakar bat virus (DBV). **e** Dengue virus (DENV). **f** Japanese encephalitis virus (JEV). Maps were generated using species distribution data from IUCN^12^, and BirdLife International and NatureServe^60^

Virus group and independent virus-specific models were used to predict probabilities for species to host flaviviruses, given data on known hosts and their respective ecological traits. Known hosts for each modeled zoonotic flavivirus, along with model-based predicted probabilities are shown in Supplementary Data file 3. The predicted probabilities for species for all the models were not highly correlated with any of the geographical traits of species indicating that model predictions were not geographically biased towards any regions of human outbreaks (Supplementary Figure 11). Out of 112 predicted host species (top 5% of species) for YFV and ZIKV (Group 1), 21 were primate species. These species on an average had 20% probability (SD = 17.0) for being a host for YFV and ZIKV. Nine of the 21 primate species predicted have not yet been detected with Group 1 viruses by confirmatory tests such as virus isolation, PCR, or validated serology (PRNT). Among the nine predicted new hosts, only the Guinea baboon (*Papio papio*) has had YFV previously detected by serology (Supplementary Data file 3)^13^. Species from Rodentia (4% mean probability, SD = 6.3, *n* = 97) showed significantly lower probabilities for being YFV and ZIKV hosts compared to Primates (Mann–Whitney–Wilcoxon *P* < 0.005, Supplementary Figure 12). The geographical ranges of predicted host species for Group 1 viruses were more widely distributed than the geographical ranges of serologically-confirmed and antigenically-confirmed positive species and showed a higher host diversity in Africa, Asia, and South America (Fig. 3). Areas in North America and Europe indicate the distributions of predicted rodent species as hosts for YFV and ZIKV, but rodent species had very low probabilities, compared to primate species in other regions. When geographical distributions of species were weighted by model-predicted YFV and ZIKV host probability values, the probability map showed hotspots with high probabilities for YFV and ZIKV sylvatic hosts in South Asia and mid-eastern Africa (South Sudan, Ethiopia, Kenya, and Somalia), and a small hotspot in the eastern coast of South America (Fig. 4).

The model predicted a total of 708 novel hosts for WNV, SLEV, and USUV, which have 254 currently recognized hosts (WNV = 194 hosts, SLEV = 72 hosts, and USUV = 65 hosts). Only species from the order Charadriiformes (12% mean probability, SD = 10.0, *n* = 95) showed a significantly different probability of hosting Group 2 viruses than species from the order Accipitriformes (39% mean probability, SD = 31.0, *n* = 40, Mann–Whitney–Wilcoxon Bonferroni-adjusted *P* < 0.005) and species from Strigiformes (38%, SD = 29.7, *n* = 19, Mann–Whitney–Wilcoxon Bonferroni-adjusted *P* = 0.01, Supplementary Figure 12). Orders that showed high predicted mean probabilities for being hosts for WNV, SLEV, or USUV were Cathartiformes (56%, SD = 36.4, *n* = 4), and Columbiformes (34%, SD = 28.7, *n* = 13). Of non-avian species, *Equus ferus* (order Perissodactyla) was in the top 5% of predicted hosts, with a probability of 8% for being a host for Group 2 viruses. None of the primates species were in the top 5% of predicted species even though *Macaca sylvanus* is a known host for WNV^14,15^ and *Ateles paniscus*, and *Sapajus apella* have been detected positive for SLEV^15,16^ (Supplementary Data file 3). Overall, two regions, North America and central Europe, showed high species richness of predicted hosts (including correctly identified known hosts) of Group 2 viruses compared to other regions (Figs. 3,  4). When the model for WNV, SLEV, and USUV was run by relabeling species as positive only when they were found positive by PCR or virus isolation, the model predicted higher mean probabilities for species from the orders Accipitriformes, Anseriformes, Carnivora, Charadriiformes, Galliformes, Passeriformes, and Rodentia (Mann–Whitney–Wilcoxon Bonferroni-adjusted *P* < 0.05). The changes in the probabilities were due to changed number of Orders included in the modeling procedure as some orders dropped out from the model as they only had species positive by PRNT (Supplementary Table 2). Predicted species in the alternate model with species confirmed only by PCR or virus isolation, revealed hotspots in the same geographical region of North America (Supplementary Figure 13).

The model for TBEV predicted 494 hosts in addition to the already recognized 75 hosts for this virus (Supplementary Data file 3). Only species from the order Passeriformes (19% mean probability, SD = 27.6, *n* = 156) showed a significantly higher probability of hosting TBEV than species from order Charadriiformes (4% mean probability, SD = 13.0, *n* = 75, Mann–Whitney–Wilcoxon Bonferroni-adjusted *P* = 0.04, Supplementary Figure 12). Mean probabilities for other Orders were not significantly different from each other (Mann–Whitney–Wilcoxon Bonferroni-adjusted *P* > 0.05, Supplementary Figure 12). The geographical distribution of these species showed high species diversity clusters across Europe and Russia (Fig. 3), with similar hotspots when geographical distributions were weighted for the predicted host probabilities (Fig. 4).

Results for the Group 3 (RBV, ENTV, and DBV) model indicated that top 5% of species with the highest predicted probabilities included 42 bat species that have not yet been detected positive for Group 3 flaviviruses. Mean predicted probabilities for hosting Group 3 viruses for families within Chiroptera were not significantly different from each other (Mann–Whitney–Wilcoxon Bonferroni-adjusted *P* > 0.05), but leaf-nosed bat species (*Phyllostomidae*) had the highest mean probability of 26% (SD = 24.2, *n* = 10, Supplementary Figure 12). Additionally, model results showed two geographical clusters with high predicted host diversity: one extending across central sub-Saharan Africa to the southeastern coast of Africa, and the second encompassing the Neotropic region and Central Americas (Figs. 3,  4).

The model for DENV predicted 173 host species, of which 139 are new, potentially unrecognized host species. The average probability for being a host for DENV among species in orders Didelphimorphia (mean = 18%, SD = 26.4, *n* = 16), Chiroptera (mean = 9%, SD = 14.2, *n* = 75), Rodentia (mean = 6%, SD = 11.3, *n* = 53), Primates (mean = 5%, SD = 2.9, *n* = 12), and Perissodactyla (mean = 3%, SD = 0.9, *n* = 13,) were not statistically different from each other (Mann–Whitney–Wilcoxon Bonferroni-adjusted *P* > 0.05, Supplementary Figure 12). Predicted species for DENV showed high, combined host species richness in the neotropical region stretching from the Central American tropics in the north to the northern Bolivian and Paraguayan regions in the south, covering all of Brazil (Figs. 3, 4).

The JEV model predicted 408 host species, of which 388 would be new hosts. Their distribution showed high species richness in Southeast Asia, central Europe, and Australia (Figs. 3,  4). Order Cetartiodactyla (mean = 3%, SD = 7.0, *n* = 45,) showed significantly higher average mean predicted probabilities than Passeriformes (mean = 2%, SD = 7.0, *n* = 135, Mann–Whitney–Wilcoxon Bonferroni-adjusted *P* = 0.048) and Primates (mean = 2%, SD = 3.0, *n* = 12, Mann–Whitney–Wilcoxon Bonferroni-adjusted *P* = 0.023). Avian orders Columbiformes (mean = 5%, SD = 13.8, *n* = 12), mammalian orders Perissodactyla (mean = 7%, SD = 18.4, *n* = 8), Chiroptera (mean = 1%, SD = 2.9, *n* = 185), and Eulipotyphla (mean = 1%, SD = 1.1, *n* = 11) showed similar mean probabilities for being hosts for JEV (Mann–Whitney–Wilcoxon Bonferroni-adjusted *P* > 0.05 Supplementary Figure 12).

## Discussion

The recent expansion of diseases such as ZIKV into Asia-Pacific regions and the Americas in 2016^17^, WNV in North America in 1999^2^, and the persistent global disease burden caused by YFV, DENV, and JEV^18^, emphasize the need to identify animal species capable of hosting flaviviruses and perpetuating human cases by sylvatic transmission. We evaluate current knowledge of host species to enhance prediction of potential new sylvatic hosts of flaviviruses in order to address important gaps in current knowledge based solely on wildlife surveillance efforts. Models highlight important features of sylvatic hosts and the macro-ecological factors underlying variation in the propensity of flaviviruses to infect certain vertebrate species. Regions highlighted with a high number of predicted wildlife hosts have heightened risk of spillover of flaviviruses because we suspect there is a higher likelihood of suitable sylvatic hosts that could maintain the virus in nature. These maps can be used to guide surveillance efforts in the event of an outbreak to verify wild reservoir species and obtain needed data on epidemiological factors related to transmission of viruses within host species and animal–human interfaces and/or vectors involved in transmission. Hierarchical clusters of flaviviruses based on similarity in host propensity resulted in groups which are part of the same flavivirus sero-complexes. For example, RBV and DBV are part of the Rio Bravo virus group, and WNV, USUV, and SLEV are part of the Japanese encephalitis virus group. Hence, while the clusters are comprised of viruses that are known to be phylogenetically similar, we demonstrate their ecological similarities as well. Whereas previous studies have used similar machine learning approaches to predict novel hosts for a single taxa group^19,20^, our modeling approach enabled evaluation of macro-ecological patterns shared by viruses from ecologically diverse viral genus across all mammal and bird species.

Life history traits such as host body mass and metabolic rates were influential in models for multiple flavivirus groups. Hosts with higher metabolic rates, especially bats and rodents that generally have a more fast-paced life history strategy, have been associated with a propensity to host a higher number of zoonotic agents including viruses, bacteria, and protozoa^19,20^. It has been hypothesized that the cost of a fast-paced life history reduces investments in the development of innate immunity, allowing species to host more pathogens^21^. Higher body temperature and metabolic rates of bats are also hypothesized to aid the ability of these species to host a wide range of pathogenic infections^22^. Our models indicate a similar trend among flaviviruses hosts, especially for YFV, ZIKV, and JEV. For all stratified models, flavivirus hosts had heavier weight than non-host vertebrate species. Even though taxonomical orders showed variation in the mean predicted probabilities, none of the taxonomical orders were found to have significantly higher probabilities for hosting flaviviruses in stratified models, except for primates which were found to have a significantly higher probability as YFV and ZIKV hosts. Life history traits and host ecology were more related to the susceptibility of species to flavivirus infection and could play an important role in the coevolution of hosts and flaviviruses.

Most flaviviruses are critically dependent on climatic conditions favoring recruitment and dispersal of their vectors and are likely to emerge or re-emerge in new areas due to projected climate change^23,24^. Travel of infected people and vectors has also enabled the emergence and re-emergence of flaviviruses in regions with suitable climate and vector distribution. The presence of a large number of susceptible hosts, as well as an increase in suitable environmental conditions related to climate change, could allow vectors to thrive and aid in establishing sylvatic cycles in newly predicted regions^24^. Our analyses indicate areas at increased risk of sustained sylvatic transmission with the ecological ingredients that could favor flavivirus transmission, even in previously naïve regions. Results indicate a heightened risk of establishing sylvatic cycles of JEV in Europe and southern Australia, where host species with potential to maintain viral circulation exist. Similarly, predicted host species in Southeast Asia could facilitate establishment of sylvatic cycles of YFV and ZIKV. Therefore, these regions with high host diversity should inform on future flavivirus surveillance and outbreak preparedness. Historically, YFV successfully spilled back into sylvatic cycle in South America after its introduction in the 17th and 18th centuries^25^. On the other hand, established sylvatic cycles of DENV has not yet been detected despite the endemicity of this virus in South America. For emerging flaviviruses such as ZIKV, there is growing concern for the establishment of sylvatic cycles due to spillback transmission from humans to wildlife in geographical regions predicted with high ZIKV host diversity^26^. Maintenance of these viruses in non-vertebrate hosts pose a great hurdle for disease control efforts, where public health measures such as vaccines could control disease transmission in urban settings, but sylvatic cycles could continue to be a source of spillover to populations in rural areas^26^. Moreover, sylvatic circulation of flaviviruses could alter primate population dynamics and contribute to extinction risks in vulnerable primate populations as has been evident for YFV-related primate declines in South America with outbreaks that have decimated populations of howler monkeys (*Alouatta spp*.)^27^.

Macro-ecological traits related to the geographical distribution of potential hosts of flaviviruses were highlighted in all models. Species in tropical and subtropical regions showed an overall trend of higher likelihood for hosting flaviviruses due to which hotspots of higher host richness for JEV, DENV, ZIKV, YFV, and for bat-specific flaviviruses were seen across tropical regions of Southeast Asia, South America and Africa. These regions are biodiverse and coincide with high human density, recent urbanization, conversion of land for agriculture, and deforestation—all of which can lead to increase in frequency of contact between humans and wildlife, thus increasing chances of viral spillover to humans^28^. Geographical features such as the distribution area of species have been previously found to be important in determining the likelihood of rodent and bat species carrying zoonotic agents^19,20^. Species with widespread distributions include commonly found peri-urban species, which have frequent interactions with humans that can lead to spillover at high-risk animal–human transmission interfaces. The significance of wider geographical distribution area, human population and livestock density, and land use patterns may indicate a generalized host trait pattern towards synanthropic species.

Following the first discovery of ZIKV in 1947 in a sentinel rhesus macaque in Uganda^29^, efforts to recognize potential natural wildlife reservoirs for ZIKV have been limited. As of 2017, the World Health Organization categorizes Southeast Asia as a region with active human transmission of ZIKV. However, evidence of ZIKV in Southeast Asian primates to date is limited to detection of exposure in Bornean orangutans (*Pongo pygmaeus*) from Eastern Sabah, Malaysia in 1996–1998^30,31^. Our model results predict additional host candidates, *Macaca fascicularis*, and *Macaca nemestrina*, which should be evaluated further for their potential to maintain ZIKV and their ability to pose ongoing spillover risk to humans.

Human cases of yellow fever have never been reported in Asia, but the potential for an outbreak with sylvatic maintenance YFV exists, as appropriate vectors are present if the virus is introduced^32^. Moreover, our models identified species with a high probability for hosting both YFV and ZIKV in South Asia, including three primate species (Fig. 3). Among these is the widely distributed *Macaca mulatta*, commonly used as an animal model in laboratory experiments^29^. Another species with a high probability for hosting YFV and ZIKV in this region is the gray langur (*Semnopithecus entellus*), which has yet to be found positive for either YFV or ZIKV. To date, evidence for ZIKV in wildlife in South Asia is limited to serological detection (complement fixation test) in three rodent species, the bandicoot rat (*Bandicota bengalensis*), Indian gerbil (*Tatera indica*), and Indian desert gerbil (*Meriones hurrianae*)^33^. However, recent detection of human cases with a local strain of ZIKV indicates there is sustained transmission of the virus within the region^34^, emphasizing a need for wildlife surveillance in this region.

The geographical distribution of predicted host probabilities for YFV and ZIKV in South America indicate lower probabilities within the Amazonian rainforests and higher probabilities around the forest perimeter. Suitability risk maps for human outbreaks of YFV are also known to follow a similar geographical distribution^35^, indicating comparable risk for both humans and wildlife and intermingling of the sylvatic cycle with the urban cycle of YFV. Species in this region with high probabilities for hosting YFV and ZIKV include several species of howler monkeys (*Alouatta caraya, Alouatta gauriba*, and *Alouatta sara*). These species are known to be sentinels for YFV outbreaks, as populations of these species often suffer die-offs due to YFV infection in advance of detection in nearby human populations^6,27^. Our model also predicts a howler monkey species in Central America, *Alouatta pigra*, which has not yet been detected positive with YFV or ZIKV. South and Central America, which have active concurrent ZIKV and YFV transmission, should be a focus for further investigation to understand the potential conservation implications of co-circulation of both YFV and ZIKV in these susceptible primate populations.

The Group 2 model indicated two regions of high host diversity for WNV, USUV, and SLEV, in North America and in Central Europe (Figs. 3,  4). After the introduction of WNV in North America, the virus caused high mortality in corvids and has been documented to cause significant reductions in the populations of other passerines^36,37^. Species with high probability for hosting Group 2 viruses include five corvid species from North America and the critically endangered Californian condor (*Cathartidae* family). Certain species, especially Accipitriformes and Anseriformes, are expected to be important in maintaining and spreading Group 2 viruses via migration.

Competent reservoirs for TBEV are known to be small forest mammals, insectivores, and livestock species, but the role of passerines and their ticks in the transmission of the infection is not well understood, even though multiple species have been detected positive^38^. Louping-ill virus (LIV), which is known to be highly homologous^39^ and shares similar biogeography to TBEV, was also found to share similar mammalian hosts (LIV has been detected in only one bird species, *Lagopus lagopus*)^38^. The geographical distribution of potential hosts for bat flaviviruses (RBV, ENTV, and DBV) when compared to predicted bat hosts for filoviruses shows similar hotspots in central Africa, but varying distribution in Southeast Asia^19^. Along with being restricted to bats, these three viruses do not have any recognized vector to date.

Dengue virus is known to have a distinct sylvatic cycle and has been detected in sentinel primate species^40^. To date, DENV has been detected via confirmatory tests (PRNT, PCR, or virus isolation) only in macaques (*Macaca nemestrina)* in Thailand^41^. The model for DENV identified eleven new Primate hosts out of which only *Macaca fascicularis*^42^ and *Pongo pygmaeus*^30^ have been previously detected serologically positive. Other instances of detection of DENV in primates are limited to Old World monkeys by serological tests with poor specificity to DENV (*Chlorocebus sabaeus*, *Erythrocebus patas*^42^, *Presbytis melalophos*, *Trachypithecus cristatus*^43^, and *Macaca sinica*^44^). Our model results indicate that marsupials (*Didelphidae* family) and bats from families *Phyllostomidae*, *Vespertilionidae*, *Molossidae*, and *Pteropodidae* could be probable candidate reservoirs in the Americas while in Asia results point towards bats as likely hosts. Field studies are needed to elucidate the potential roles for primates and bats in the transmission and maintenance of DENV, especially in Asia.

Japanese encephalitis virus has been circulating endemically in Eastern and Southeastern Asia. We identified potential passerine hosts from regions in Southeast Asia and Europe. The high probability regions in the Indian subcontinent predicted in this study coincide with regions known to have reported cases of JEV^45^. Concern for the establishment of a JEV endemic cycle in Europe is rising due to the introduction of this virus by movement of people and the increasing suitability of the climate to sustain vectors, especially given the detection of JEV in a *Culex pipiens* mosquito in northern Italy^46^. Based on our predictions, potential host species that could sustain JEV transmission exist in Europe, including some of the most common passerines, such as the Barn Swallow (*Hirundo rustica*) and the Carrion Crow (*Corvus corone*).

Results presented here highlight significantly important species and regions of the world that should be prioritized for sylvatic surveillance of flaviviruses. Surveillance targets should be considered for their role as reservoir, amplifying, or dead-end hosts, as well as their ability to produce a level of viremia that is likely to propagate transmission. For example, WNV cases in humans are associated with the proliferation of virus in avian amplification hosts, but not with infected equines or humans because the latter two species produce little viremia^47^. Similarly, vectors and ecological conditions that are likely to facilitate transmission to humans will greatly impact the propensity of predicted species in hotspot regions to perpetuate sylvatic cycles. The host diversity hotspots shown here include both the wintering and breeding grounds of migratory birds, especially in case of WNV, SLEV, and USUV which inlcude temperate regions that are not likely to favor year round transmission because of reduced abundance of vectors and shorter summer seasons. Even with well-informed targets for surveillance, field-based detection of flaviviruses in wild animal hosts requires a large-scale effort with a low probability of detecting positives, especially given the very short duration of infection for most flaviviruses. Sero-surveys, which are becoming increasingly specific, have been used to detect positive animals during ongoing outbreaks (e.g., of 4–6% for WNV in wild birds^47^). Detection of YFV in animals is often triggered by mortality events in New World monkeys^27,48^. Aside from outbreak investigations, comprehensive longitudinal sampling efforts for sylvatic host detection has been uncommon due to logistical hurdles.

We demonstrate that macroecological host traits can be used to predict undiscovered hosts of zoonotic viruses over diverse vertebrate taxa including birds and mammals. Our analyses identified potential regions with high host diversity that could be prioritized for flavivirus surveillance, and we were able to narrow down avian and mammalian targets with potentially important roles in sylvatic transmission. Large-scale ecological and climate changes will lead to shifts in the distribution of both hosts and vectors, with direct implications for spillover risk of flaviviruses from host species. As global surveillance efforts continue to generate more data on the distribution of wildlife hosts and as new hosts are discovered, the modeling framework presented here can be used to anticipate regions with flavivirus emergence vulnerability and better inform on the impact of sylvatic transmission on global health.

## Methods

### Datasets

A list of known viruses from the *Flavivirus* genus was generated using the NCBI taxonomy database^49^ and the 2016 taxonomy report of the International Committee on Taxonomy of Viruses (ICTV)^10,50^. A systematic literature review of peer-reviewed articles published through September 2016 was completed using PubMed taxonomy IDs to collect data on all vertebrate hosts of thirty-five zoonotic flaviviruses covering two-thirds of all viruses in the genus *Flavivirus*^10^ globally. We assumed a flavivirus to be zoonotic if it was known to be reported in humans as well as in a non-human vertebrate host, despite the absence of specific evidence of transmission from animals to humans. Similarly, GenBank entries were downloaded and used to search for hosts^51^. Other databases such as Global Infectious Diseases and Epidemiology Network (GIDEON)^52^, the Centers for Disease Control Arbovirus catalog^53^, the World Animal Health Information System OIE, the World Health Organization, ProMED^54^, and the virus pathogen database and analysis resource (ViPR)^55^, were also queried.

Experimental infections of animals in laboratory settings were excluded to collect data only on hosts of flaviviruses which can get infected via natural infection. For all flaviviruses, positive serological detection except with the plaque reduction neutralization test (PRNT) in a host was not considered conclusive, due to poor test specificity and cross-reactivity with other flaviviruses. Such hosts were classified as “suspected hosts” unless serologic detection was followed by a confirmatory test. Detection of a virus by virus isolation, molecular assay (PCR), or plaque reduction neutralization tests (PRNT) was considered confirmatory^56^. Hierarchical clustering of viruses according to their confirmed hosts in different orders was done using the Bray–Curtis dissimilarity index to identify clusters of flaviviruses sharing similar sylvatic hosts.

For all species of mammals and birds, life history, physiological, and ecological trait data were collected from multiple sources. Taxonomic classification, conservation status, habitat utilization, and population temporal trend data were collated from the International Union for Conservation of Nature (IUCN) using the letsR R package^57^. Data for body temperature, metabolic rate^58^, body mass, and foraging attributes such as diet, foraging strata, and foraging time were also included^59^. Habitat distribution (global range) of terrestrial mammals and birds were downloaded from IUCN^12^ and BirdLife International^60^, respectively. Shapefiles describing geographical range of species are suitable for calculating the distributions of bio-climatic, and diversity-related species traits and for generating presence/absence matrices for the species distribution^57^. These distribution shapefiles were used to calculate geographical bounds, distribution areas, and geographical centroids for the species distribution.

Global distribution data for bioclimatic variables, mammalian and avian diversity and anthropogenic variables were collected from multiple sources^61–63^. For each of the global raster datasets, we calculated zonal statistics for all species using their geographical habitat distributions. A complete list of variables, their explanations, sources, and calculated statistics can be found in Supplementary Table 3 and Supplementary Table 4.

The interactions of vectors and hosts, biting preferences of ticks and mosquitoes, and vector competence are all critical parameters in maintaining virus circulation in a sylvatic environment. Since these data are not available, we included a variable describing the spatial distribution of vectors within the geographic range of a host. A systematic literature review was also done to identify flavivirus vectors. Distribution data for 18 of the most common known mosquito vectors and eight of the most common known tick vectors were procured from VectorMap^64^, which included vector species that have been reported to carry 28 out of 35 flavivirus species evaluated in this analysis (Supplementary Table 5). Mosquito and tick species distribution rasters represented probabilities for the occurrence of the species, with values ranging from 0 to 1. These rasters were then superimposed and summed together to generate two separate cumulative distribution maps of mosquito vectors and tick vectors. These cumulative distribution rasters were finally used to calculate zonal statistics for all bird and mammalian species. Missing data were imputed using *K*th-nearest neighbor imputation methods using five nearest neighbors.

To control for potential reporting bias due to imbalanced sampling efforts to collect and report data on flavivirus hosts, we included the number of primary publications in PubMed using a MeSH term search for the avian and mammalian species as a proxy for study efforts, assuming that surveillance efforts for vertebrate species will be proportional to the number of citations available for them. This approach also allowed us to account for geographical variation in the surveillance as species are geographically distributed. Supplementary Figure 14 shows the geographical variation in the PubMed hits used in the generic model to account for sampling biases. Many of the large-scale surveillance programs target probable species candidates, and hence sufficient sampling effort among animals that have tested negative for a virus was rare. While knowledge of true negative species would have facilitated model training, ascertainment of species status as non-hosts (i.e., unable to harbor flavivirus infection naturally) was not feasible. Thus, we choose a conservative approach of identifying species that share similar macro-ecological traits as known hosts. For modeling purposes, we generated a binary code for each mammal and bird species (1, if the species is currently known to host any flavivirus and 0, if not, Supplementary Data file 3), generating a baseline for prediction based on known species, which will be iteratively improved following the discovery of new flaviviruses hosts with appropriate field data.

### Analysis and modeling

We applied generalized boosted regression tree (BRT) methods using the aforementioned twenty-nine host traits as predictors for binary code generated for species to host a flavivirus (generic *Flavivirus* model). Such models are useful here, as boosting improves the predictive performance of many weak models substantially, can fit complex nonlinear relationships, and can automatically handle interaction effects between predictors^11^. For this analysis, data were partitioned into training and holdout sets (80% of observations used for training, 20% used as holdout) and relative sizes of positive samples were kept similar in both the sets (Supplementary Table 1). For model building, we used a 10-fold cross-validation method to avoid overfitting for tuning model parameters. Using this method, training data were randomly partitioned into ten, equal-sized subsamples and each subsample was retained once as testing data, while the rest of the data was used to train the model. We used the gbm step function from R package gbm^65^, to assess the optimal number of boosting trees, as described in Elith et al.^11^ The BRT model generates additive models in a step-wise manner while optimizing the differentiable loss function. At each stage, a regression tree was fit on a Bernoulli loss function. The holdout dataset was then used for external validation of the model performance. Relative influence of host traits was estimated using the equation developed by Friedman for tree-based methods^66^ and is based on the number of times a variable is selected for splitting, weighted by the squared improvement to the model as a result of each split averaged over all trees^11^. For assessment of the robustness of the methodology with respect to reporting bias, we reran the generic model by excluding the host factor related to the number of primary publications in PubMed.

To evaluate the clustering of hosts due to phylogenetic relatedness within taxonomic groups, we included dummy categorical variables for all host families related to the model. Along with the generic flaviviruses model, which was developed to explore macroecological traits of Flavivirus sylvatic hosts, individual models were developed for flaviviral clusters and flaviviruses that did not cluster with any other virus (TBEV, JEV, and DENV). The stratified models were developed to explore the virus-specific host patterns and to minimize biases related to variability in research effort among viruses. Bias towards the inclusion of more well-studied hosts is likely to influence results of the generic model. Binary responses for these models were based on whether a host species was known to carry any of the viruses within each virus group. Virus-specific models were restricted only to species from taxonomical orders with confirmed hosts for those viruses (Supplementary Table 4). To evaluate model robustness to species classifications, we developed a second model for group 2 viruses (WNV, SLEV, and USUV) with hosts reclassified as positive only if they had been detected positive by PCR or virus isolation (and not considering species found positive by PRNT).

We explored 90th and 95th percentiles of our model predictions and probability values that maximized the true positive rate + true negative rate for the holdout datasets to identify the threshold that best highlighted species as probable hosts for flaviviruses. Known geographic distributions^12,60^ of predicted species in the 95th percentile of model predictions were plotted to identify regions with a higher diversity of predicted hosts. To represent model-predicted probabilities with species distributions, we assigned predicted probability values for each host species to the raster layer representing the geographical ranges of the hosts and summed the probabilities across all the raster layers to generate maps showing hotspots with high predicted host probability.

### Code availability

All R code used for developing models and python code for generating figures needed to replicate and evaluate these analyses are provided at 10.5281/zenodo.1482000.

## Supplementary information

Supplementary Information

Description of Additional Supplementary Files

Supplementary Data 1

Supplementary Data 2

Supplementary Data 3

## Data Availability

Data collected during the study including virus host associations, citations and GenBank accessions number are provided in Supplementary Data file 1.
