# Supplementary material for: Predicting wildlife reservoirs and global vulnerability to zoonotic Flaviviruses
Source: Nat Commun. 2018 Dec 21;9:5425. doi: 10.1038/s41467-018-07896-2 (PMC6303316; doi:10.1038/s41467-018-07896-2)
Supplement: Supplementary file 1 — Supplementary Information [file 41467_2018_7896_MOESM1_ESM.pdf]

Predicting wildlife reservoirs and global vulnerability to zoonotic Flaviviruses

Pandit et al.

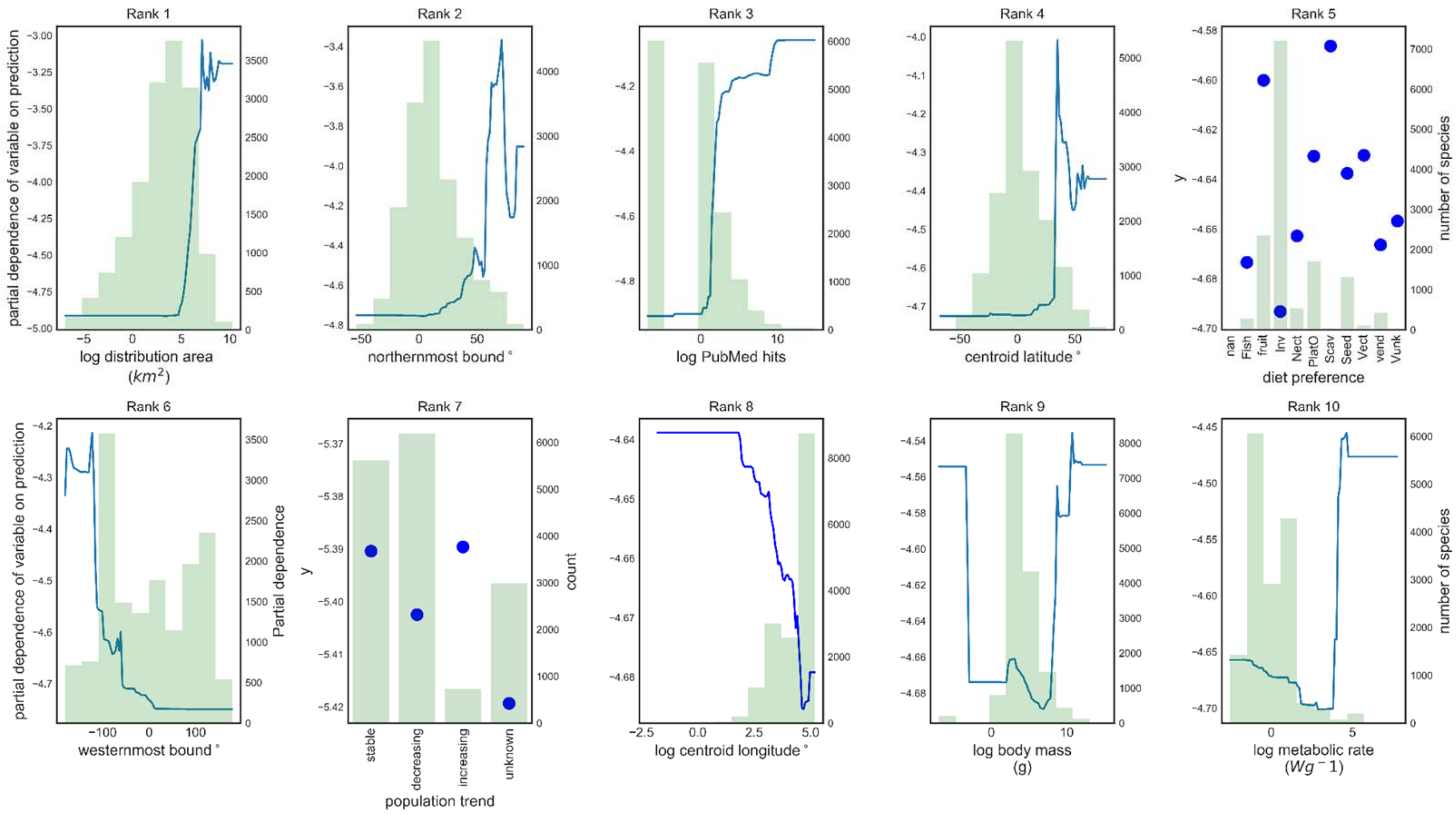

Supplementary Figure 1. Trait profile of zoonotic Flavivirus-positive species. Partial dependence plots of the top 10 predictor traits from the generalized boosted regression trees model, arranged in order of variable importance. Lines (continuous variables) and dots (categorical variables) indicate the partial dependence of the variable on the prediction and the histogram indicates the distribution of the variable in the model data. Note: Diet preference: Fish: Fish; fruit: Fruit/drupes; Inv: Invertebrates including aquatic invertebrates; Nect: Nectar pollen, plant exudates, gums; PlantO: Other plant material; Seed: Seeds; Vect: Reptiles, snakes, amphibians; vend: Mammals, Birds; Vunk: Vertebrates-general or unknown

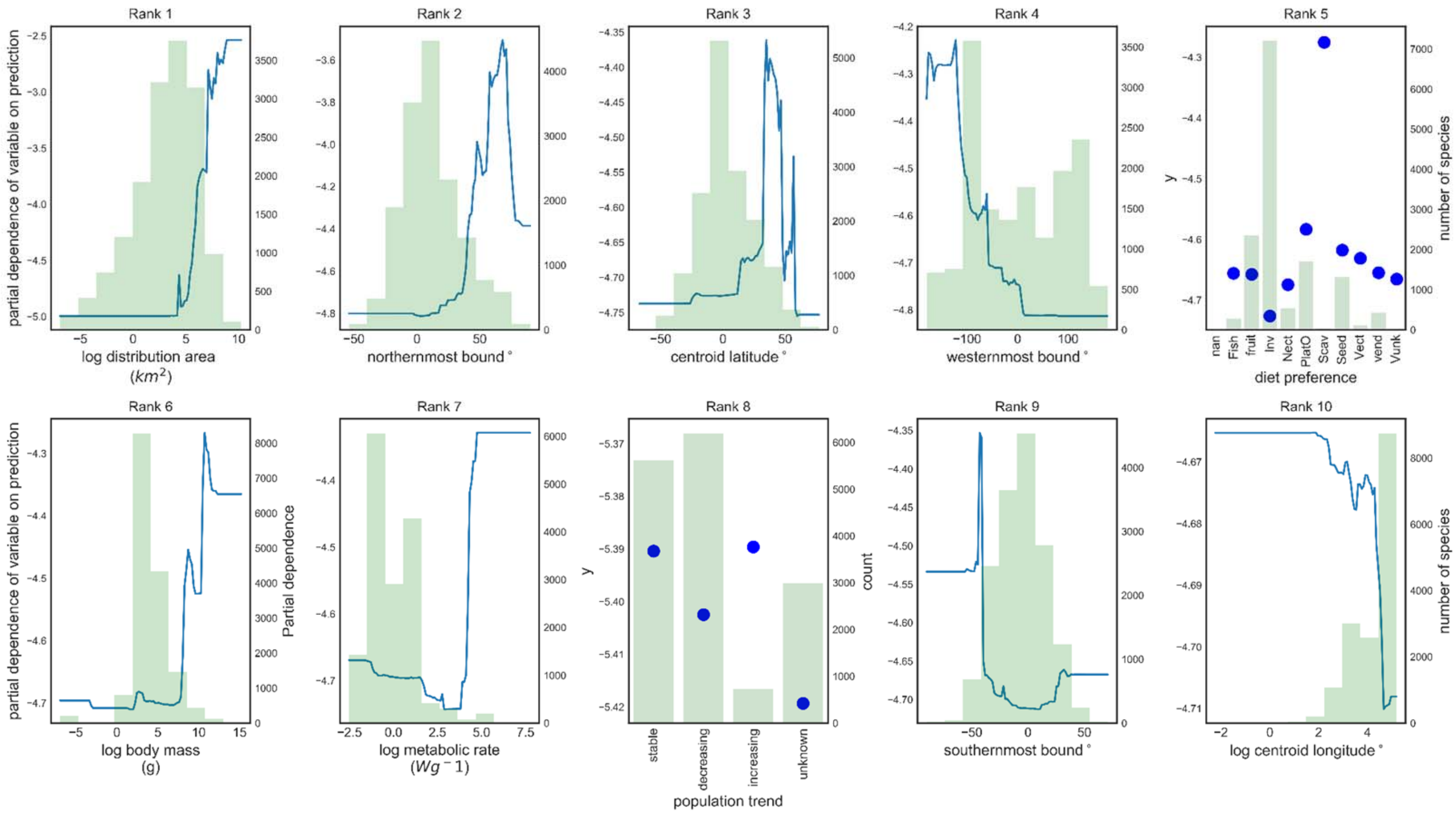

Supplementary Figure 2. Trait profile of zoonotic Flavivirus-positive species using a model excluding the variable PubMed hits. Partial dependence plots of the top 10 predictor traits from the generalized boosted regression trees model, arranged in order of variable importance. Lines (continuous variables) and dots (categorical variables) indicate the partial dependence of the variable on the prediction and the histogram indicates the distribution of the variable in the model data. Note: Diet preference: Fish: Fish; fruit: Fruit/drupes; Inv: Invertebrates including aquatic invertebrates; Nect: Nectar pollen, plant exudates, gums; PlantO: Other plant material; Seed: Seeds; Vect: Reptiles, snakes, amphibians; vend: Mammals, Birds; Vunk: Vertebrates-general or unknown

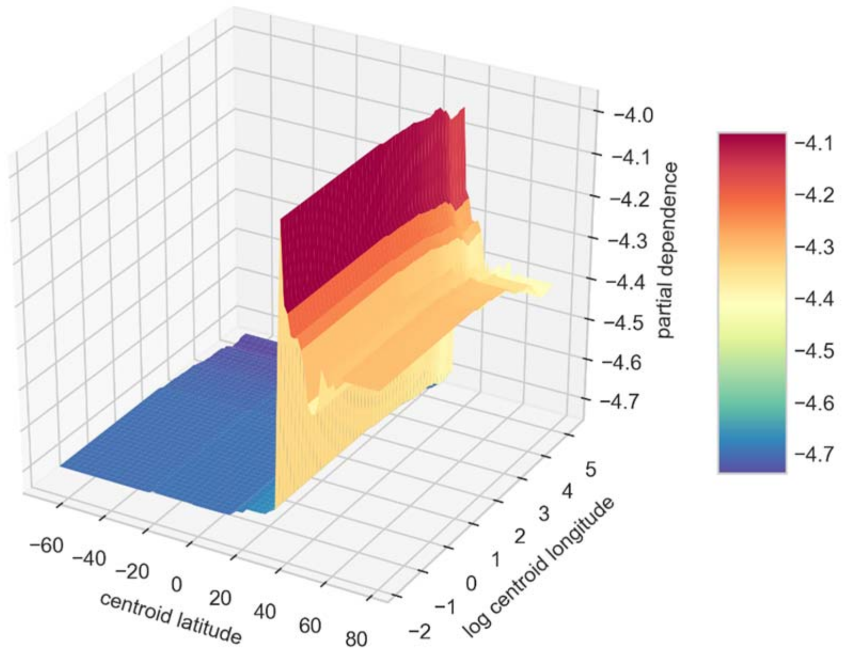

Supplementary Figure 3. Partial dependence and interaction of latitude and longitude centroids of vertebrate species for being flaviviral host in the generic Flavivirus model.

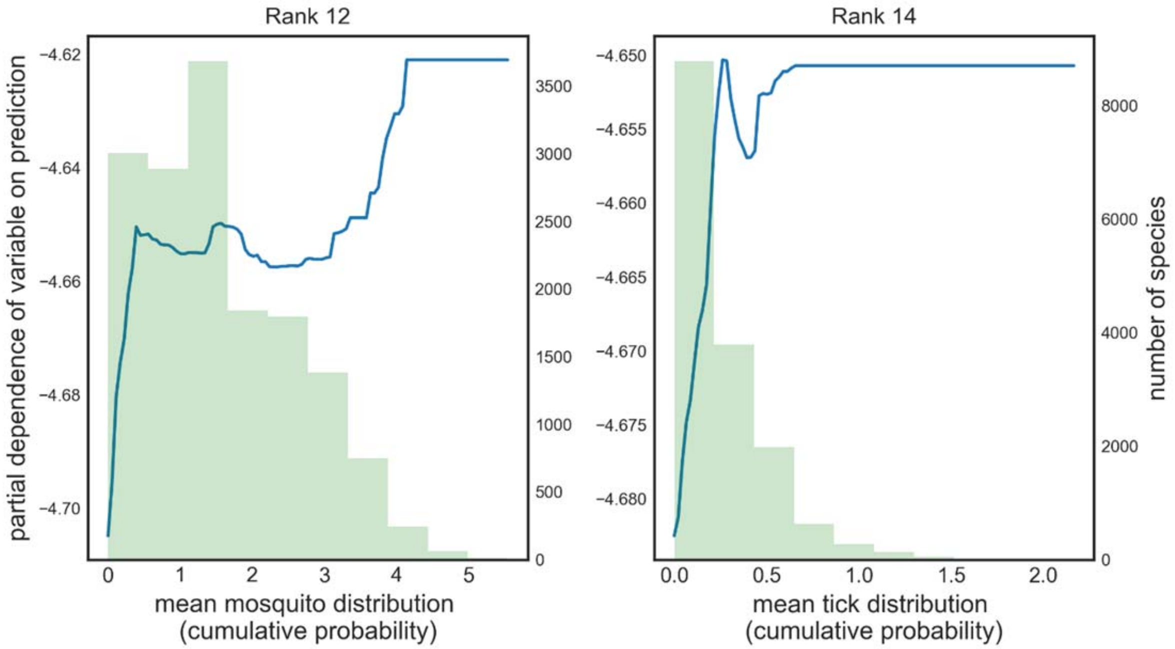

Supplementary Figure 4. Partial dependence of variables related to the distributions of mosquito and tick vectors for the generic Flavivirus model.

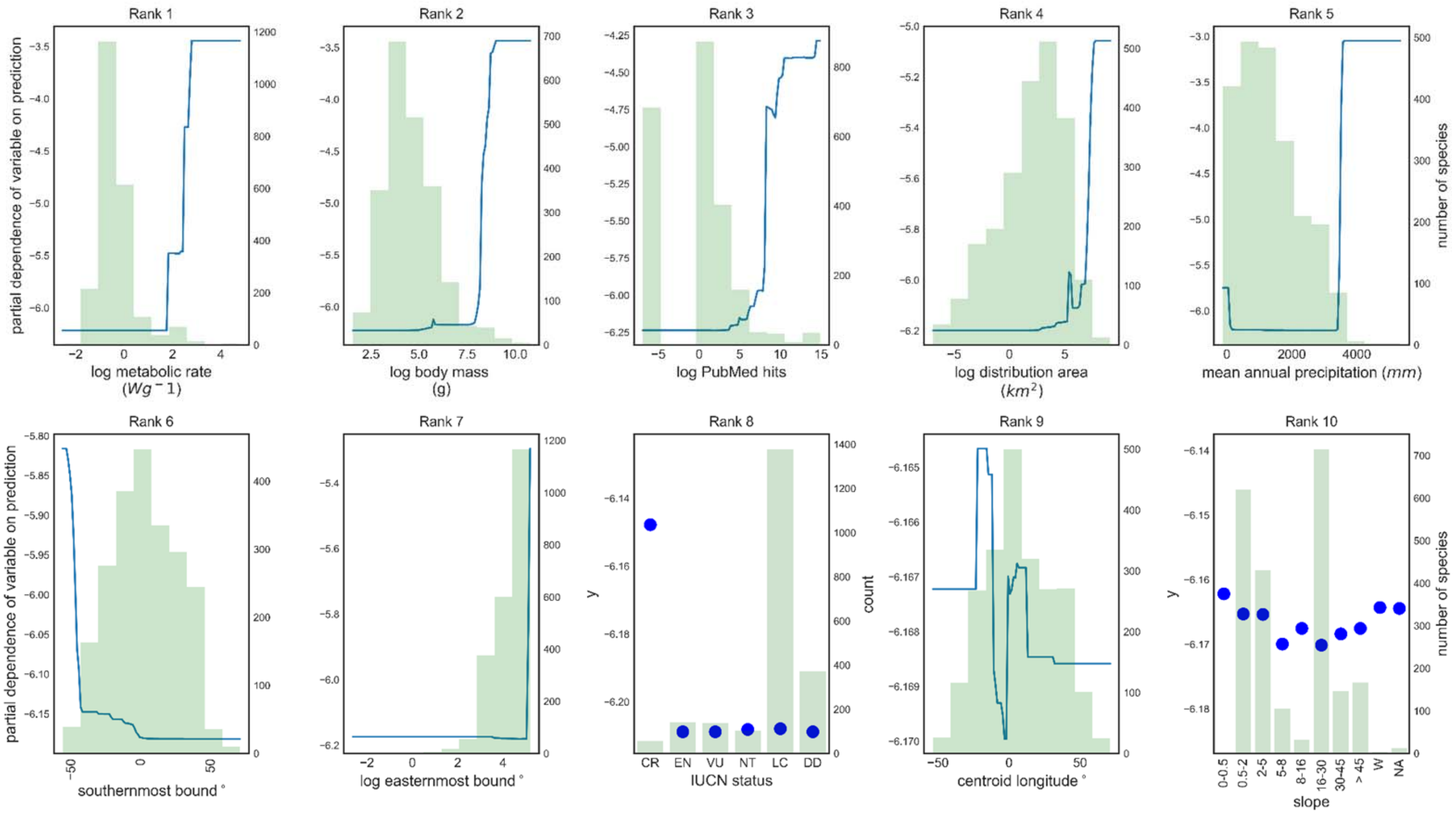

Supplementary Figure 5. Trait profile of YFV- and ZIKV-positive species. Partial dependence plots of the top 10 predictor traits from the generalized boosted regression trees model, arranged in order of variable importance. Lines (continuous variables) and dots (categorical variables) indicate the partial dependence of the variable on the prediction and the histogram indicates the distribution of the variable in the model data.

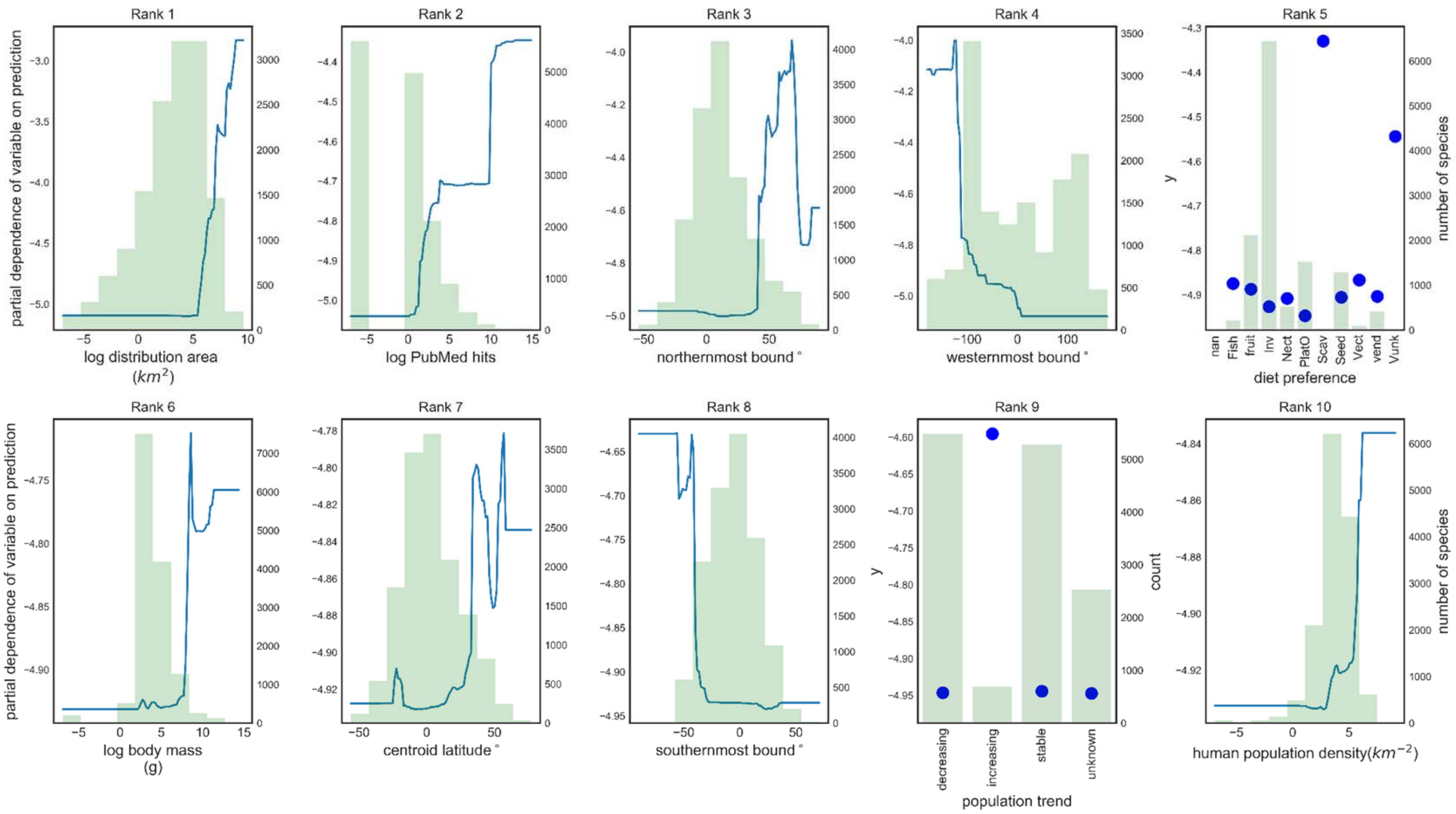

Supplementary Figure 6. Trait profile of WNV, SLEV, and USUV positive species: Partial dependence plots of the top 10 predictor traits from the generalized boosted regression trees model, arranged in order of variable importance. Lines (continuous variables) and dots (categorical variables) indicate the partial dependence of the variable on the prediction and the histogram indicates the distribution of the variable in the model data. Note: Diet preference: Fish: Fish; fruit: Fruit/drupes; Inv: Invertebrates including aquatic invertebrates; Nect: Nectar pollen, plant exudates, gums; PlantO: Other plant material; Seed: Seeds; Vect: Reptiles, snakes, amphibians; vend: Mammals, Birds; Vunk: Vertebrates-general or unknown

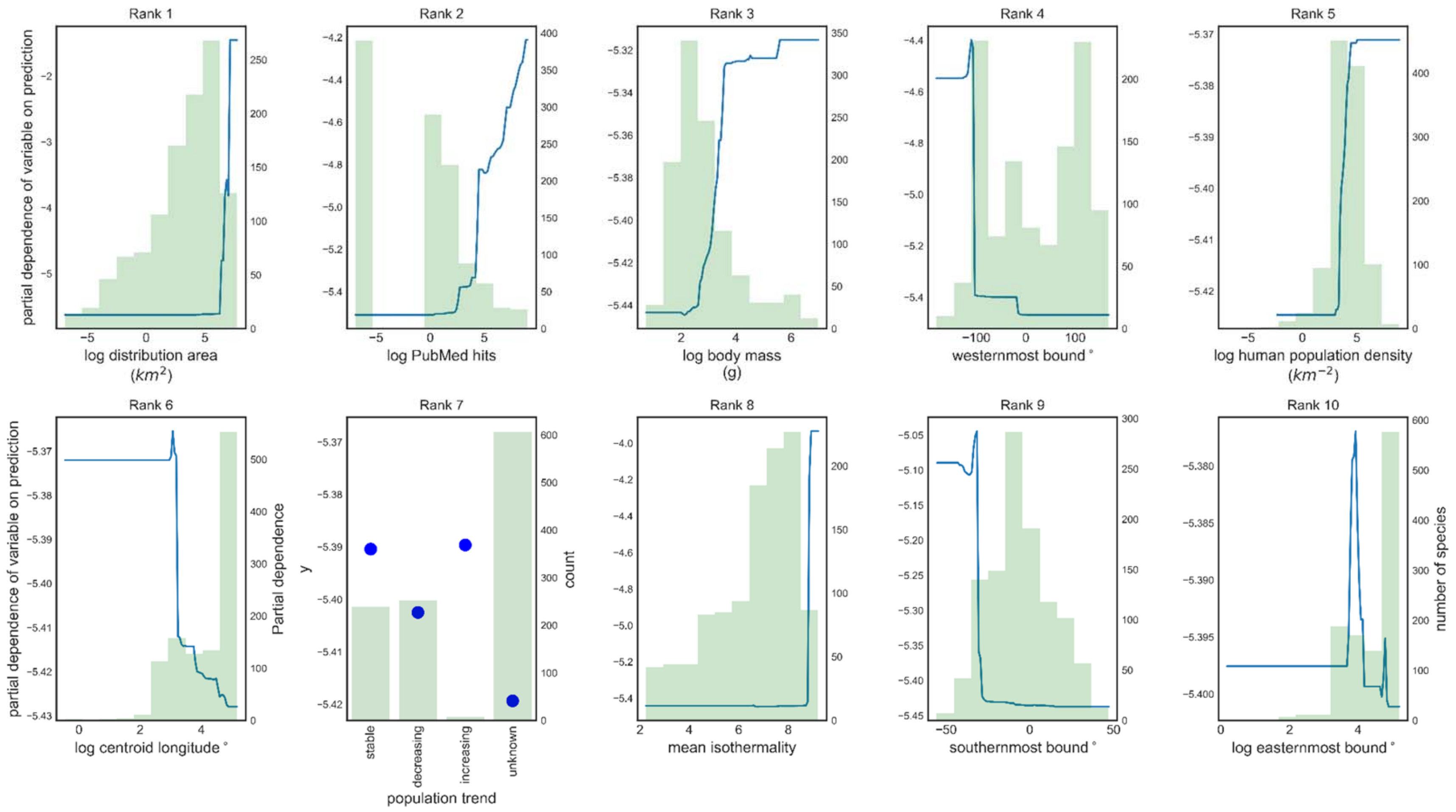

Supplementary Figure 7. Trait profile of RBV, ENTV, and DBV positive species. Partial dependence plots of the top 10 predictor traits from the generalized boosted regression trees model, arranged in order of variable importance. Lines (continuous variables) and dots (categorical variables) indicate the partial dependence of the variable on the prediction and the histogram indicates the distribution of the variable in the model data.

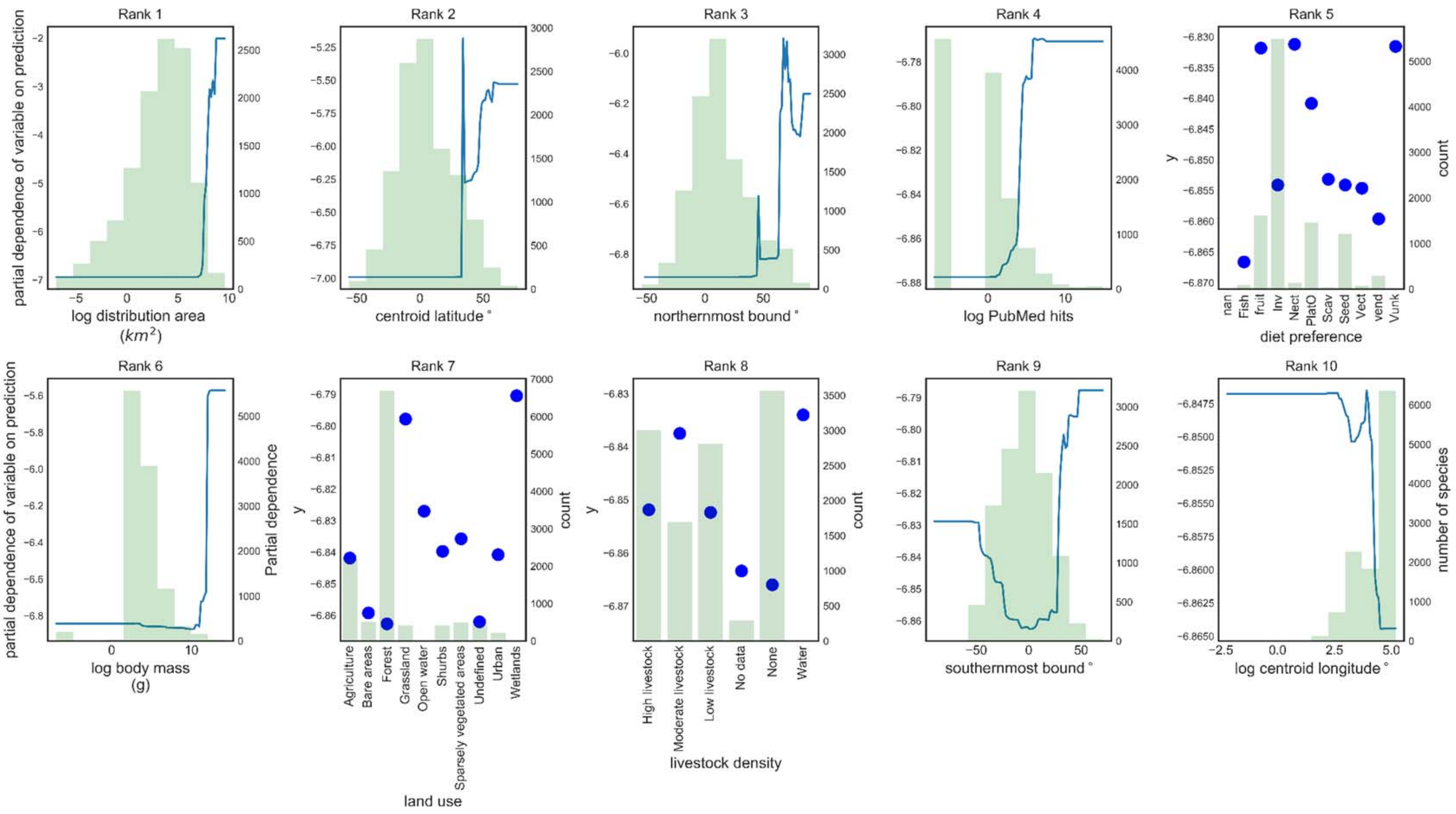

Supplementary Figure 8. Trait profile of TBEV positive species. Partial dependence plots of the top 10 predictor traits from the generalized boosted regression trees model, arranged in order of variable importance. Lines (continuous variables) and dots (categorical variables) indicate the partial dependence of the variable on the prediction and the histogram indicates the distribution of the variable in the model data. Note: Diet preference: Fish: Fish; fruit: Fruit/drupes; Inv: Invertebrates including aquatic invertebrates; Nect: Nectar pollen, plant exudates, gums; PlantO: Other plant material; Seed: Seeds; Vect: Reptiles, snakes, amphibians; vend: Mammals, Birds; Vunk: Vertebrates-general or unknown

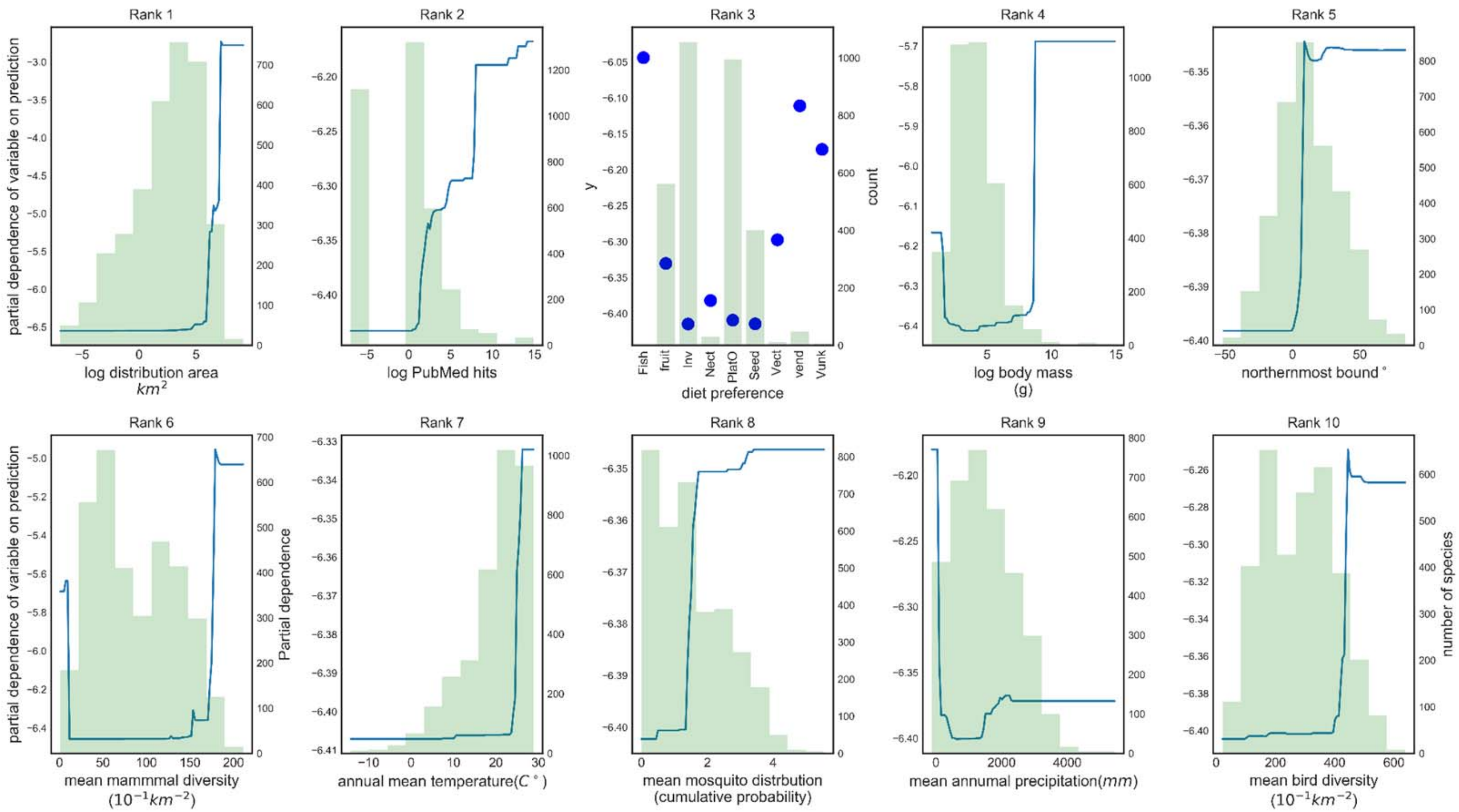

Supplementary Figure 9: Trait profile of DENV positive species. Partial dependence plots of the top 10 predictor traits from the generalized boosted regression trees model, arranged in order of variable importance. Lines (continuous variables) and dots (categorical variables) indicate the partial dependence of the variable on the prediction and the histogram indicates the distribution of the variable in the model data. Note: Diet preference: Fish: Fish; fruit: Fruit/drupes; Inv: Invertebrates including aquatic invertebrates; Nect: Nectar pollen, plant exudates, gums; PlantO: Other plant material; Seed: Seeds; Vect: Reptiles, snakes, amphibians; vend: Mammals, Birds; Vunk: Vertebrates-general or unknown

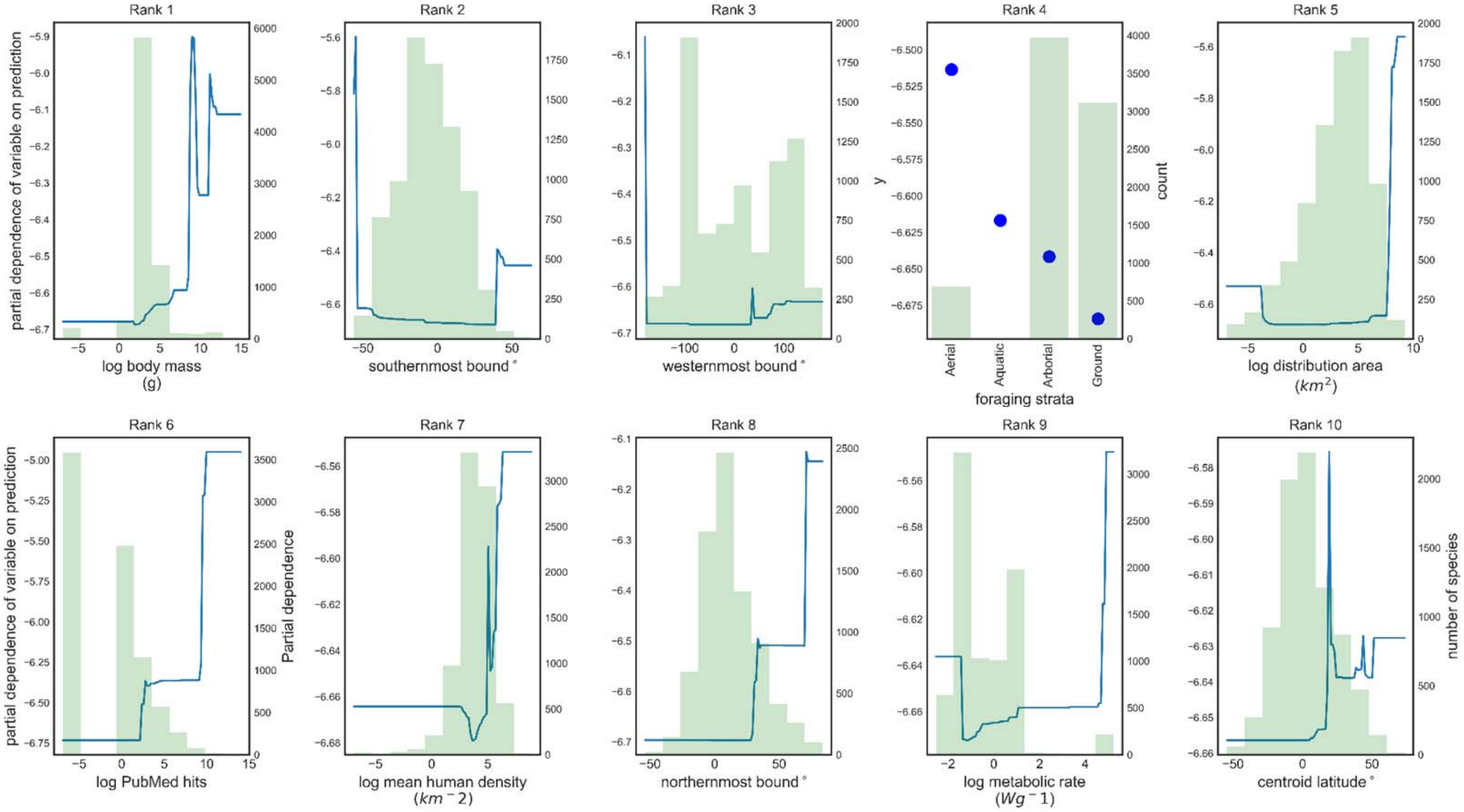

Supplementary Figure 10. Trait profile of JEV positive species. Partial dependence plots of the top 10 predictor traits from the generalized boosted regression trees model, arranged in order of variable importance. Lines (continuous variables) and dots (categorical variables) indicate the partial dependence of the variable on the prediction and the histogram indicates the distribution of the variable in the model data.

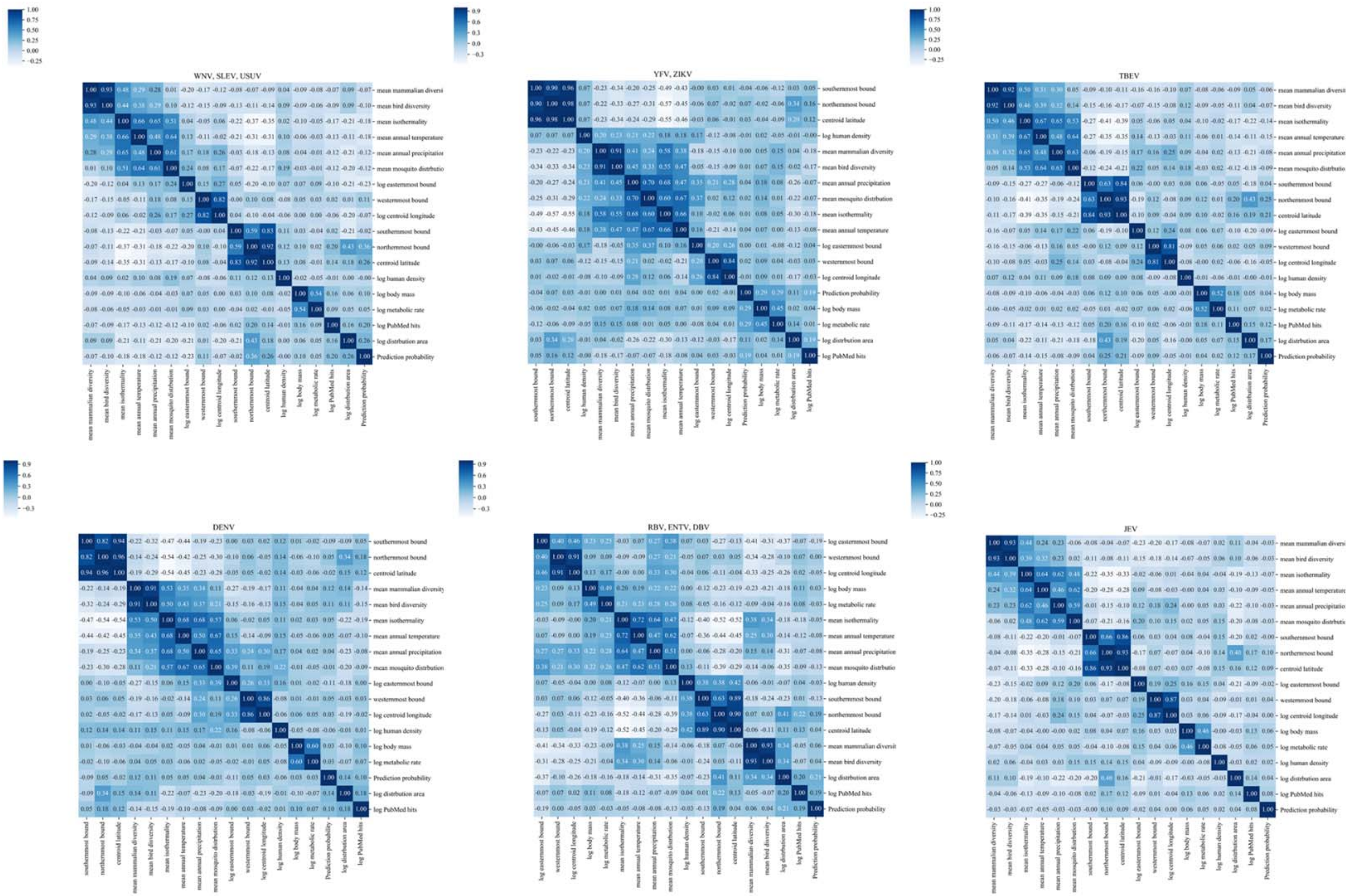

Supplementary Figure 11: Hierarchically-clustered heatmap showing correlations between continuous host traits used as predictor variables and predicted probability for mammalian and avian species.

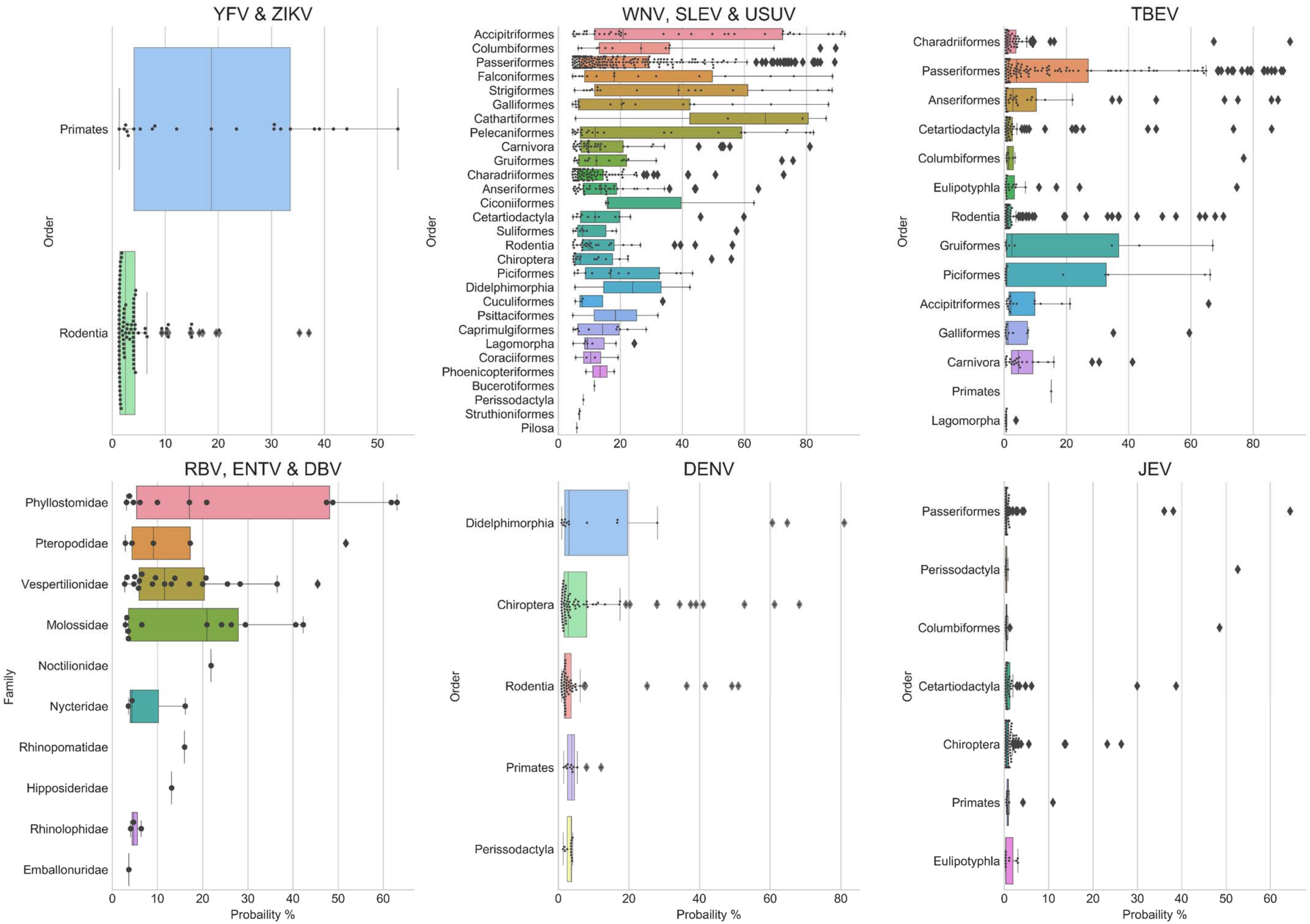

Supplementary Figure 12. Model-predicted probabilities. Boxplots show the distribution of model-predicted probabilities for species from modeled orders; dots show data points for species and diamonds show outlier species.

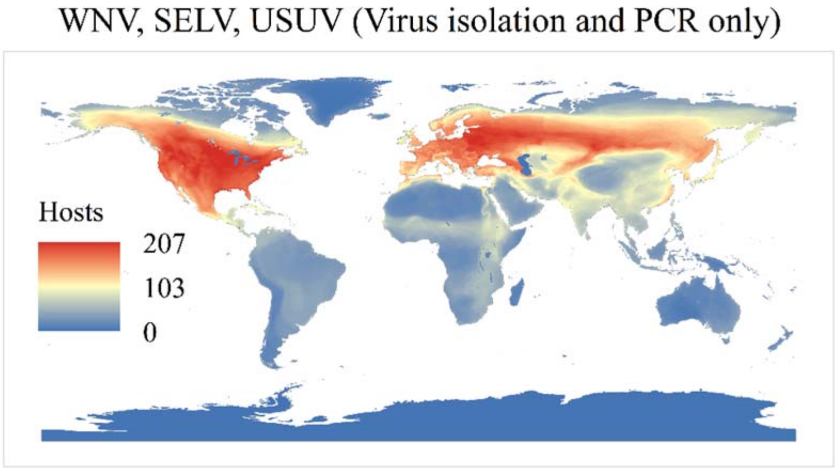

Supplementary Figure 13: Overlapping geographical ranges of model predicted hosts in the 95th percentile of probability for WNV, SLEV and USUV by relabeling positive hosts only when they are found positive by PCR or virus isolation. Map was generated using species distribution data from IUCN[1], and BirdLife International and NatureServe[2].

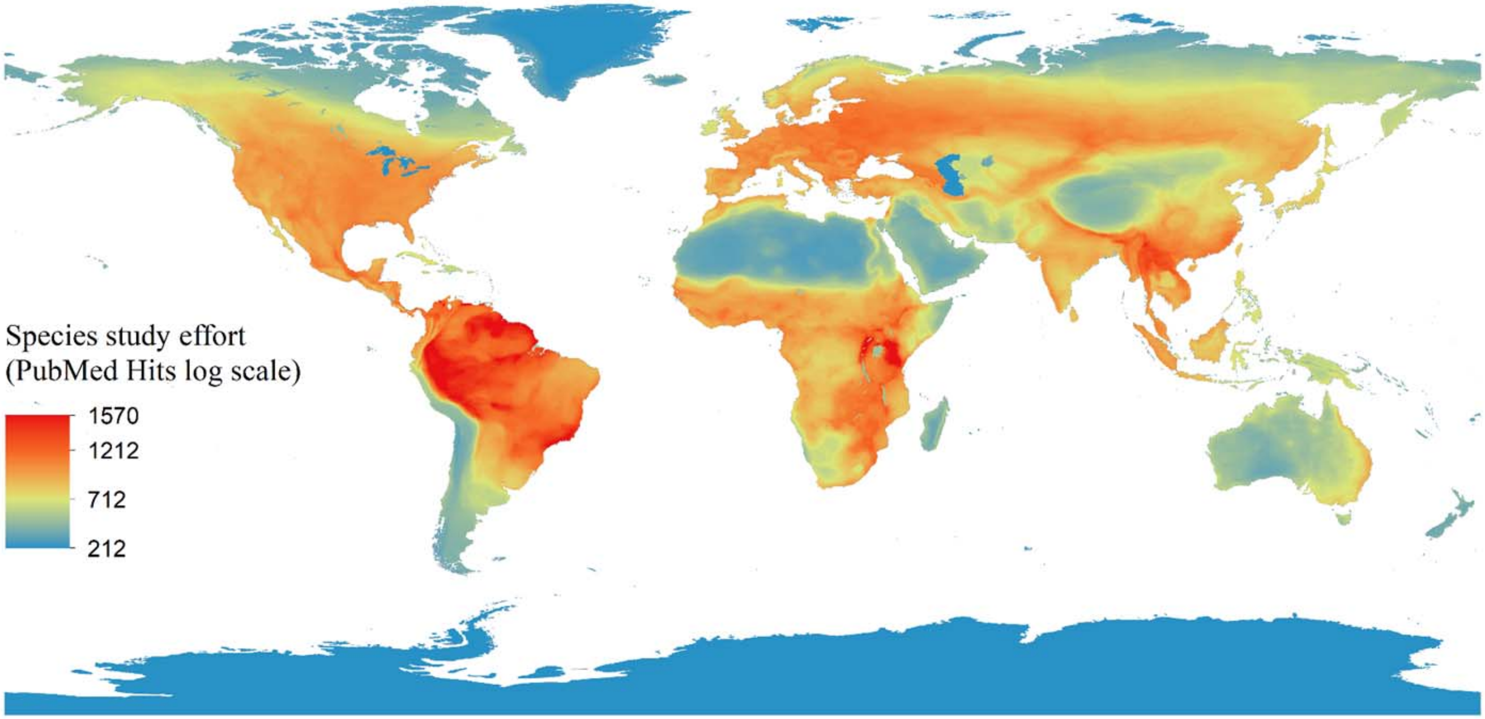

Supplementary Figure 14: Geographical distribution of study effort shown by overlapping geographical ranges of all the mammalian and avian species and their associated studies reported in PubMed Central database. Map was generated using species distribution data from IUCN[1], and BirdLife International and NatureServe[2].

Supplementary Tables

Supplementary Table 1. Model parameters and the optimal number of trees for different models

Model	***Postive samples***		***Total samples***		***Optimal Number of trees***	***cv AUC score (SE)***	***Holdout AUC***
	Training	***Holdout***	***Training***	***Holdout***			
Generic	*305*	*76*	*12236*	*3056*	*9250*	*0.906 (0.011)*	*0.923*
Generic (without PubMed hits)	*305*	*76*	*12236*	*3056*	*11200*	*0.894 (0.01)*	*0.924*
Group 1: YFV, ZIKV	*11*	*3*	*1774*	*444*	*1400*	*0.944 (0.03)*	*0.975*
Group 2: WNV, SLEV, USUV	*203*	*51*	*11112*	*2778*	*5850*	*0.903 (0.008)*	*0.938*
Group 2: WNV, SLEV, USUV (PCR/virus isolation)	*118*	*30*	*11197*	*2799*	*6450*	*0.924 (0.011)*	*0.892*
TBEV	*62*	*15*	*9038*	*2260*	*4400*	*0.978 (0.004)*	*0.972*
Group 3: RBV, ENTV, DBV	*13*	*3*	*871*	*218*	*1700*	*0.929 (0.031)*	*0.662*
DENV	*18*	*5*	*2740*	*685*	*2200*	*0.909 (0.041)*	*0.877*
JEV	*16*	*4*	*6501*	*1625*	*1250*	*0.834 (0.046)*	*0.992*

Note: *tree complexity: 5, learning rate: 0.001, bag fraction: 0.5, cv: 10*

Supplementary Table 2. Taxonomic Orders known to have confirmed species for viruses from each virus group that are used for predictions in respective models

**Virus Groups**	**Orders**
Group 1: YFV, ZIKV	*Primates, Rodentia*
Group 2: WNV, SLEV, USUV	*Accipitriformes, Passeriformes, Charadriiformes, Strigiformes, Galliformes, Primates, Anseriformes, Pelecaniformes, Chiroptera, Rodentia, Cetartiodactyla, Pilosa, Bucerotiformes, Carnivora, Caprimulgiformes, Falconiformes, Piciformes, Columbiformes, Gruiformes, Cathartiformes, Suliformes, Perissodactyla, Phoenicopteriformes, Cuculiformes, Psittaciformes, Coraciiformes, Ciconiiformes, Struthioniformes, Lagomorpha, Didelphimorphia*
Group 2: WNV, SLEV, USUV (PCR/Isolation only)	*Accipitriformes, Passeriformes, Charadriiformes, Strigiformes, Galliformes, Primates, Anseriformes, Pelecaniformes, Chiroptera, Rodentia, Cetartiodactyla, Bucerotiformes, Carnivora, Caprimulgiformes, Falconiformes, Piciformes, Columbiformes, Gruiformes, Cathartiformes, Suliformes, Perissodactyla, Phoenicopteriformes, Psittaciformes, Coraciiformes, Ciconiiformes, Struthioniformes, Didelphimorphia*
TBEV	*Rodentia, Cetartiodactyla, Eulipotyphla, Primates, Lagomorpha, Carnivora, Passeriformes, Piciformes, Galliformes, Charadriiformes, Anseriformes, Gruiformes, Accipitriformes, Columbiformes*
Group 3: RBV, ENTV, DBV	*Chiroptera*
DENV	*Chiroptera, Didelphimorphia, Perissodactyla, Primates, Rodentia*
JEV	*Cetartiodactyla, Columbiformes, Eulipotyphla, Perissodactyla, Chiroptera, Passeriformes, Primates*

***Note**: Blue: avian orders*

Supplementary Table 3. Feature dataset used in the generalized boosted regression model for predicting flavivirus potential hosts

Model Category	***Variable***	***Variable Type***	***Description***	***Source***
Conservation Status	*IUCN status*	*categorical*		[1]
	Population Trend	*categorical*		[1]
Ecology	*Habitat breadth*	*numeric*	*Number of habitats*	[3]
	Diet preference	*categorical*	*predominant diet type*	[4]
	Foraging strata	*categorical*	*Foraging Strata*	[4]
	Activity time	*categorical*	*diurnal-nocturnal*	[4]
Physiology	*Metabolic rate (W)*	*numeric*	*metabolic rate*	[4]
	Body temperature (°C)	*numeric*	*body temperature*	[4]
	Body mass (g)	*numeric*	*body mass*	[4]
Distribution	*Centroid (X, Y)*	*numeric*	*centroids of distribution shapefile*	
	Northernmost bound	*numeric*	*Y maximum value*	-
	Southernmost bound	*numeric*	*Y minimum value*	-
	Easternmost bound	*numeric*	*X maximum value*	-
	Westernmost bound	*numeric*	*X minimum value*	-
	Distribution Area (km^{-2})	*numeric*	*area of the distribution shapefile*	-

Supplementary Table 4. Model variables calculated from global raster datasets

Model Category	***Variable***	***Variable Type***	***Description***	***Source***	***Statistic***
Bio-Climatic	*Annual precipitation (mm)*	*numeric*		[5]	*mean*
	Annual Mean Temperature (°C)	*numeric*		[5]	*mean*
	Isothermality	*numeric*	*comparison of daily difference to the yearly difference*	[5]	*mean*
	Slope	*categorical*		[6]	*mode*
Diversity	*Mammal diversity (n×10 km^{-2})*	*numeric*	*mammalian diversity in the range of species*	[7]	*mean*
	Bird diversity (n×10 km^{-2})	*numeric*	*avian diversity in the range of species*	[7]	*mean*
Anthropogenic	*Human population density (km^{-}*	*numeric*	*Human population diversity in the range of species*	[6]	*mean*

variables	^{2}*)*				
	Crops	*categorical*		[6]	*mode*
	Livestock density	*categorical*		[6]	*mode*
	Land use	*categorical*		[6]	*mode*
Vectors	*Mosquitoes*	*numeric*	*additive distribution of mosquito vectors (probability × 10)*	[8]	*mean*
	Ticks	*numeric*	*additive distribution of tick vectors (probability × 10)*		*mean*

Supplementary Table 5: Known Flavivirus mosquitoes and tick vectors and viruses carried by these vectors

Vector	***Number of viruses***	***Viruses***						
Aedes aegypti	*7*	*DENV*	*ILHV*	*SLEV*	*WSLV*	*WNV*	*YFV*	*ZIKV*
Culex pipiens	*6*	*JEV*	*SLEV*	*TMUV*	*USUV*	*WNV*	*ZIKV*	
Culex tritaeniorhynchus	*6*	*BAGV*	*DENV*	*JEV*	*TMUV*	*WSLV*	*WNV*	
Aedes albopictus	*5*	*DENV*	*MVEV*	*USUV*	*WNV*	*ZIKV*		
Aedes vexans	*5*	*JEV*	*TBEV*	*USUV*	*WSLV*	*WNV*		
Culex quinquefasciatus	*5*	*JEV*	*SLEV*	*TMUV*	*WNV*	*ZIKV*		
Aedes africanus	*5*	*BOUV*	*WSLV*	*WNV*	*YFV*	*ZIKV*		
Culex annulirostris	*4*	*JEV*	*KUNV*	*MVEV*	*WNV*			
Psorophora ferox	*4*	*ILHV*	*ROCV*	*SLEV*	*WNV*			
Mansonia uniformis	*4*	*JEV*	*WSLV*	*WNV*	*ZIKV*			
*Ixodes persulcatus**	*4*	*LIV*	*OMSKV*	*TBEV*				
Culex univittatus	*3*	*USUV*	*WSLV*	*WNV*				
Culex vishnui	*3*	*JEV*	*TMUV*	*WNV*				
Culex pseudovishnui	*3*	*JEV*	*KUNV*	*TMUV*				
*Dermacentor marginatus**	*3*	*OMSKV*	*TBEV*	*WNV*				
Culex bitaeniorhynchus	*3*	*JEV*	*MVEV*	*WNV*				
Aedes scapularis	*3*	*ILHV*	*ROCV*	*SLEV*				
Sabethes chloropterus	*3*	*ILHV*	*SLEV*	*YFV*				
*Hyalomma marginatum**	*3*	*LIV*	*TBEV*	*WNV*				
Aedes luteocephalus	*3*	*WSLV*	*YFV*	*ZIKV*				
Anopheles hyrcanus	*3*	*JEV*	*WNV*					
*Amblyomma variegatum**	*2*	*WNV*	*YFV*					
*Rhipicephalus muhsamae**	*2*	*WSLV*	*WNV*					
*Ixodes ricinus**	*2*	*LIV*	*TBEV*					
*Dermacentor reticulatus**	*2*	*OMSKV*	*TBEV*					
*Amblyomma cajennense**	*1*	*CPCV*						

**Tick species.*

Note: *APOIV: Apoi virus, BAGV: Bagaza virus, BANV: Banzi virus, BOUV: Bouboui virus, CPCV: Cacipacore virus, DBV: Dakar bat virus, DENV: Dengue virus, ENTV: Entebbe bat virus, IGUV: Iguape virus, ILHV: Ilheus virus, JEV: Japanese encephalitis virus, KFDV: Kyasanur forest virus, KUNV: Kunjin virus, LIV: Louping-ill virus, MODV: Modoc virus, MVEV: Murray Valley encephalitis virus, OMSKV: Omsk hemorrhagic fever virus, RBV: Rio*

Bravo virus, ROCV: Rocio virus, SLEV: St. Louis Encephalitis virus, TBEV: Tickborne encephalitis virus, TMUV: Tembusu virus, UGSV: Uganda S virus, USUV: Usutu virus, WNV: West Nile virus, WSLV: Wesselsbron virus, YFV: Yellow fever virus, ZIKV: Zika virus.

Supplementary References

1. IUCN. The IUCN Red List of Threatened Species. Version 2017-1 edn (2017).

2. BirdLife International and Nature Serve. Bird species distribution maps of the world. BirdLife International, Cambridge, UK and NatureServe, Arlington, USA. (2015).

3. Vilela B, Villalobos F. letsR: a new R package for data handling and analysis in macroecology. *Methods Ecol Evol* **6**, 1229-1234 (2015).

4. Wilman H, Belmaker J, Simpson J, de la Rosa C, Rivadeneira MM, Jetz W. EltonTraits 1.0: Species□level foraging attributes of the world's birds and mammals. *Ecology* **95**, 2027-2027 (2014).

5. Hijmans RJ, Cameron SE, Parra JL, Jones PG, Jarvis A. Very high resolution interpolated climate surfaces for global land areas. *Int J Climatol* **25**, 1965-1978 (2005).

6. FAO. FAO□Geonetwork. Food and Agriculture Organization of the United Nations (2017).

7. Jenkins CN, Pimm SL, Joppa LN. Global patterns of terrestrial vertebrate diversity and conservation. *Proc Natl Acad Sci U S A* **110**, E2602-2610 (2013).

8. The Walter Reed Biosystematics Unit. VectorMap. http://vectormap.si.edu/. Smithsonian Institution (2016).
