# Supplementary material for: Predicting wildlife reservoirs and global vulnerability to zoonotic Flaviviruses
Source: Nat Commun. 2018 Dec 21;9:5425. doi: 10.1038/s41467-018-07896-2 (PMC6303316; doi:10.1038/s41467-018-07896-2)
Supplement: Supplementary file 2 — Description of Additional Supplementary Files [file 41467_2018_7896_MOESM2_ESM.pdf]

Description of Additional Supplementary Files

File Name: Supplementary Data 1

Description: Data showing virus-host associations, citations and GenBank accessions numbers.

File Name: Supplementary Data 2

Description: The relative importance of variables for different models.

File Name: Supplementary Data 3

Description: Species predictions generated by all stratified generalized boosted regression models for virus groups and the labels used for training data.
